# The acute effects of joint manipulative techniques on markers of autonomic nervous system activity: a systematic review and meta-analysis of randomized sham-controlled trials

**DOI:** 10.1186/s12998-019-0235-1

**Published:** 2019-03-12

**Authors:** Mathieu Picchiottino, Charlotte Leboeuf-Yde, Olivier Gagey, David M. Hallman

**Affiliations:** 1 CIAMS, Université Paris-Sud, Université Paris-Saclay, Orsay Cedex, France; 2 CIAMS, Université d’Orléans, Orléans, France; 3 Institut Franco-européen de Chiropraxie (IFEC), Ivry-sur-Seine, France; 4 Institute for Regional Health Research, University of Southern Denmark, Odense, Denmark; 5 Centre for Musculoskeletal Research, Department of Occupational and Public Health Sciences, University of Gävle, Gävle, Sweden

**Keywords:** Autonomic nervous system, Sympathetic nervous system, Parasympathetic nervous system, High velocity low amplitude manipulation, Mobilization, Joint manipulative techniques, Systematic review, Système nerveux autonome, Système nerveux sympathique, Système nerveux parasympathique, Manipulation de haute vélocité et de faible amplitude, Mobilisation, Technique de manipulation articulaire, Revue systématique

## Abstract

**Background:**

The autonomic nervous system (ANS) interests many chiropractors and manual therapists, because joint manipulative techniques (JMT), e.g. high velocity low amplitude (HVLA) manipulations and mobilizations, appear to produce acute changes in ANS mediated physiology. The complexity of this issue justifies a systematic critical literature review.

**Objective:**

To review the literature comparing the acute changes in markers of ANS activity between JMT applied on spinal or peripheral joints and a sham procedure in healthy or symptomatic subjects.

**Method:**

We searched PsycINFO, PEDro, PubMed, Cochrane library, EMBASE, and Medline up to December 2017. We updated the search with PubMed, Cochrane library, EMBASE, and Medline including July 2018. Inclusion criteria were: randomized sham-controlled trials assessing the effect of JMT on markers of ANS activity; manually applied JMT, regardless of technique, applied on either healthy or symptomatic humans; outcome measurements recorded at baseline and repeated during and/or after interventions. Selection of articles and data extraction were performed independently by two reviewers. The quality of studies was assessed using the *Cochrane ‘risk of bias’* tool and a technical check-list. Results were reported narratively with some meta-analyses. The *Cochrane* GRADE approach was used to assess the certainty of evidence.

**Results:**

Twenty-nine of 2267 studies were included in the synthesis. Mobilizations (oscillatory technique) probably produce an immediate and short-term, bilateral increase in skin sympathetic nerve activity (reflected by an increase in skin conductance) regardless of the area treated (moderate-certainty evidence). It is uncertain whether the sympathetic arousal also explains an increase in respiratory rate (very low-certainty evidence). Our evaluation of the literature suggests that spinal sustained apophyseal glides (SNAGs) mobilization and HVLA manipulation of the spine may have no acute effect on the studied markers of ANS activity (very low- to low-certainty evidence).

**Conclusion:**

Some types of mobilizations probably produce an immediate and short-term, statistically significant increase in skin sympathetic nerve activity when compared to a sham procedure, whereas spinal SNAGs and spinal HVLA techniques may have no acute effect on the studied markers of ANS activity. No region-specific results were noted. The literature suffers from several shortcomings, for which reason we strongly suggest further research.

**Electronic supplementary material:**

The online version of this article (10.1186/s12998-019-0235-1) contains supplementary material, which is available to authorized users.

## Résumé

**Introduction:**

Le système nerveux autonome (SNA) intéresse de nombreux chiropracteurs et thérapeutes manuels car les techniques de manipulation articulaire, e.g. mobilisations ou manipulations de haute vélocité et faible amplitude (HVLA) semblent produire des changements immédiats de l’activité du SNA. La complexité de la littérature sur le sujet justifie une revue critique de la littérature.

**Objectif:**

Examiner la littérature comparant les changements immédiats d’activité du SNA entre i) différentes techniques de manipulation articulaire appliquées sur le rachis ou les articulations périphériques et ii) une procédure *placebo* chez des sujets sains ou pathologiques.

**Méthode:**

Nous avons cherché sur PsycINFO, PEDro jusqu’en décembre 2017 et sur PubMed, Cochrane library, EMBASE, Medline jusqu’en juillet 2018. Les critères d’inclusion étaient: essais randomisés contrôlés par un *placebo* évaluant l’effet d’une technique de manipulation articulaire sur l’activité du SNA, sans restriction concernant la technique ou la population, mesures de l’activité du SNA réalisées avant, pendant et/ou après l’intervention. La qualité des études était évaluée avec la grille de risque de biais de *Cochrane* et avec une grille d’évaluation technique. La sélection des études et l’extraction des données étaient effectuées indépendamment par deux chercheurs. Les résultats ont été rapportés narrativement, parfois avec des méta-analyses, en évaluant la certitude du niveau de preuve avec l’approche GRADE de *Cochrane*.

**Résultats:**

Nous avons inclus 29 études sur les 2267 trouvées. Il y a des preuves de certitude modérée que les mobilisations (avec une technique d’oscillation) produisent une augmentation bilatérale, immédiate et à court terme de l’activité sympathique cutanée, indépendamment de la région mobilisée. Nous ne savons pas si l’excitation sympathique explique une augmentation de la fréquence respiratoire. Il y a des preuves de faible certitude que la manipulation spinale (HVLA) ainsi qu’une autre technique de mobilisation spinale n’ont pas d’effet sur l’activité autonome mesurée.

**Conclusion:**

Certaines techniques de mobilisation articulaire produisent probablement une augmentation (statistiquement significative) immédiate et à court terme de l’activité sympathique cutanée comparées à une procédure *placebo*. Les manipulations spinales (HVLA) pourraient ne pas avoir d’effet immédiat sur l’activité autonome étudiée. Nous n’avons pas remarqué d’effet spécifique en fonction de la zone du traitement. Cette littérature montre des lacunes. Pour cette raison, nous suggérons (vivement) de nouvelles études.

## Introduction

Joint manipulative techniques (JMT) are commonly used to treat different musculoskeletal pain conditions by a large spectrum of therapists [1]. These treatments consist of various techniques applied either on spinal or on peripheral joints, such as different types of mobilizations and high velocity low amplitude (HVLA) manipulations. Despite their popularity among therapists and patients [1] and intensive scientific research for many years, we still do not understand how these techniques work. In fact, research has been conducted in domains such as biomechanics [2–4] and neurophysiology using outcomes related to neuromuscular response [3], pain [5], and even autonomic nervous system (ANS) activity [6–8] but without providing a definitive comprehensive model of either the effects or the underlying mechanisms of action of JMT [9].

The ANS is a major part of the nervous system, which is composed by two anatomically and functionally distinct branches, the parasympathetic and the sympathetic system. Its ultimate responsibility is to ensure the maintenance of homeostasis by regulating cells, tissues and function of organs [10]. The ANS is controlled by supraspinal centers such as the limbic system, hypothalamus, and some brainstem nuclei (e.g. the periaqueductal gray area) [10]. In general, autonomic activation can be assessed indirectly via various non-invasive markers of autonomic mediated physiology, such as skin conductance [11] and heart rate variability [12].

The possible effects of JMT on ANS activity have received considerable research attention [6–8]. However, the underlying mechanisms are still hypothetical. It has been stated that the somato-autonomic reflexes [13] are often invoked as potential mechanisms for the acute changes in autonomic mediated physiology (e.g. visceral responses) after a JMT [14]. It is worth noting that JMT seem to produce both hypoalgesia [5] and sympatoexcitation [6, 7]. Thus, it was proposed that the periaqueductal gray matter, a structure that initiates anti-nociceptive processes and autonomic regulation [15], might be activated [16]. Nevertheless, *Schmid* et al. [17] concluded that there is insufficient evidence to suggest that the responses (hypoalgesia and sympathetic arousal) might involve specific activation of this structure.

It is also interesting to note that the early concepts of chiropractic proposed that the correction of a spinal dysfunction could lead to better visceral functioning by ‘normalizing’ autonomic activity. Nowadays, it is still not unusual to find some therapists performing manipulation to improve specific visceral problems [18] and, sometimes, to do so based on the concept of the relation between the vertebrae and the anatomical organization of the ANS, as shown in the ‘Meric system’ [19, 20]. However, there is presently no obvious clinical evidence supporting the rationale for this activity [21].

Since there is a vast and fairly complicated literature on this concept, we considered it relevant to perform a systematic review on the acute effects of JMT on markers of ANS activity to provide evidence-based information, which would be helpful to both researchers and clinicians.

Several literature reviews have already explored, as a primary aim, the acute effects of JMT on autonomic mediated physiology [6–8]. In their review and meta-analysis, *Chu* et al. [6] found that joint mobilizations of the thoracic and cervical spine produced a sympatho-excitatory response in the upper extremity, similarly to *Kingston* et al. [7], who found that joint mobilizations of the spine resulted in a sympathetic excitation. *Amoroso Borges* et al. [8], in a recent review, concluded that cervical / lumbosacral and thoracic manipulations may stimulate, respectively, the parasympathetic and sympathetic nervous system. Before undertaking a new systematic review, it would be relevant to consider the scope and some methodological aspects of previous systematic work.

First, these reviews differ in the design and the quality of the included studies. *Chu* et al. [6] *and Kingston* et al. [7] included randomized controlled trials on both healthy and symptomatic subjects and assessed their quality with a PEDro scale. The quality of the papers was reported as good, thus they provided good quality evidence. The third review [8] included both randomized controlled trials and non-randomized trials with quality ranging from poor to good, assessed by the PEDro scale. Therefore, the conclusions of this last review should be interpreted with caution.

Second, in the three reviews [6–8], the quality assessment did not take into account some important aspects of the autonomic measurements, such as considering whether the experimental conditions were appropriately controlled or if the data processing was transparent and pertinent, which would limit the credibility of the results, if there were quality issues in these areas.

Third, each review [6–8] covered one particular technique (mobilizations or HVLA manipulations) applied to the spine (or to a limited area of the spine). For these reasons, it is difficult to generalize their conclusions to all types of JMT.

Finally, taking into account the large number of studies and the ambition to provide the best quality evidence concerning the effects of JMT, new systematic reviews should be based, as far as possible, on studies which compare the impact of JMT to that of a sham control. This is necessary, if the aim is to provide evidence on the difference between specific changes, induced by the supposed effective intervention, to those (positive or negative) attributable to the brain-mind responses (e.g. placebo or nocebo) [22].

In conclusion, to the best of our knowledge, the extent to which different types of JMT - applied either on the spine or the peripheral joints - acutely affect ANS mediated physiology compared to a sham procedure remains partially unknown. In addition to the previous reviews, the use of an assessment of both risk of bias and of the technical quality of the experiment, applied on randomized sham-controlled trials may give rise to new perspectives and may form the basis for interesting discussions and further research. We therefore performed a new systematic review, which included these two quality aspects (risk of bias and technical aspects).

Our aim was to review the literature, comparing the acute changes in markers of ANS activity between JMT applied on spinal or peripheral joints and a sham procedure in healthy or symptomatic subjects.

Our research question was:

What are the specific acute effects of different techniques of mobilizations and HVLA manipulations applied on spinal or peripheral joints in healthy or symptomatic subjects on markers of ANS activity?

## Method

This systematic review was performed following, when it was relevant, the Preferred Reporting Item for Systematic Reviews and Meta-Analysis PRISMA [23].

Registration number in PROSPERO: CRD42016050858. Please note that some changes were done as the review unfolded.

### Literature search

We performed a systematic literature search using the following databases: PubMed, the Cochrane library, PsycINFO, PEDro, EMBASE, and Medline from inception until December 2017. The search was updated until July 2018 with PubMed, the Cochrane library, EMBASE, and Medline.

The search strategy is shown in Table 1. It was initially used on PubMed and adapted for the other databases. The search was restricted to randomized controlled trials, clinical trials, and clinical studies on humans. We limited the search to articles reported in English or French.

**Table 1:** Search terms used

“spinal manipulative therapy” OR	“lumbar mobilization” OR	“peripheral mobilization” OR	AND	“autonomic nervous system” OR
“spinal manipulation” OR	“cervicothoracic mobilization” OR	“mobilization with movement” OR		“sympathetic nervous system” OR
“spine manipulation” OR	“thoracolumbar mobilization” OR	“Maitland mobilization” OR		“parasympathetic nervous system” OR
“thrust manipulation” OR	“lumbosacral mobilization” OR	“chiropractic” OR		“sympathetic” OR
“joint manipulation” OR	“sacroiliac mobilization” OR	“osteopathy” OR		“parasympathetic” OR
“cervical manipulation” OR	“osteopathic manipulation” OR	“manual therapy” OR		“heart rate variability” OR
“thoracic manipulation” OR	“osteopathic manipulative treatment” OR	“manipulation” OR		“skin blood flow” OR
“lumbar manipulation” OR	“chiropractic manipulation” OR	“HVLA” OR		“skin temperature” OR
“cervicothoracic manipulation” OR	“chiropractic adjustment” OR	“mobilization” OR		“skin conductance” OR
“thoracolumbar manipulation” OR	“orthopaedic manipulation” OR	“Manipulation, Osteopathic” [Mesh] OR		“blood pressure” OR
“lumbosacral manipulation” OR	“musculoskeletal manipulations” OR	“Manipulation, Chiropractic” [Mesh] OR		“heart rate” OR
“sacroiliac manipulation” OR	“spinal mobilization” OR	“Manipulation, Spinal” [Mesh] OR		“breath rate” OR
“cervical mobilization” OR	“joint mobilization” OR	“Manipulation, Orthopaedic” [Mesh] OR		“cardiovascular” OR
“thoracic mobilization” OR	“glide mobilization” OR	“Musculoskeletal Manipulations” [Mesh]		“Autonomic Nervous System” [Mesh]

### Eligibility criteria

The articles were included if they fulfilled the following inclusion criteria. The studies had to be randomized sham-controlled trials assessing the effect of a JMT on markers of ANS activity. The JMT had to be applied manually on either healthy or symptomatic humans. The outcome measurements had to be performed at baseline and repeated during and / or after the intervention(s).

We accepted studies using markers of ANS activity as outcomes such as i) skin conductance (SC) an indicator of the skin sympathetic nerve activity [11] and ii) heart rate variability (HRV) [12]. We also accepted outcome variables such as iii) heart rate and blood pressure, iv) various biochemical markers (e.g. salivary alpha amylase or plasma norepinephrine concentration), and v) pupil diameter. Finally, we accepted also other outcome variables, if they were reported as indicators of the ANS in the study e.g. vi) skin blood flow, vii) skin temperature, and viii) respiratory rate. The outcome variables used in the included studies will be discussed in the discussion section.

Studies using HVLA mechanically-assisted techniques (e.g. activator instrument) were not included in the review, as these techniques are less likely to induce joint movements.

### Selection of articles

Two of the authors independently screened and selected the relevant articles by applying the eligibility criteria on titles and abstracts. If there was a disagreement, a third investigator would arbitrate.

### Classification of articles and data extraction

Articles were categorized according to the JMT used in the experiments, i.e. HVLA manipulation and different types of mobilizations. Data extraction concerning the main features (Additional file 1), the risk of bias (Additional file 2), the technical quality (Additional file 3), and results (Additional file 4) of each trial was performed by two independent researchers.

### Overall approach to assess the evidence

The assessment of the evidence contains three steps. We first assessed two important components relating to the quality of each included study; (i) the risk of bias and (ii) the technical aspects of the experiment. These domains reflect two different quality aspects in this type of research that should be considered in the assessment of the evidence. Risk of bias tools are often used to detect errors in the overall research design (e.g. randomization process, blinding of study subjects and assessors) that can result in systematic errors. Therefore, risk of bias is different from technical quality that refers to technical requirements to obtain valid data (e.g. experiment performed under well-controlled laboratory conditions, appropriate use of testing equipment). Finally, we assessed (iii) the *overall* certainty of evidence for each outcome variable. This was done by taking into account these two quality items together with other factors (detailed below) that can affect the certainty of evidence. These three steps are described below.

### 1/ Assessment of risk of bias in included studies

#### Contents of the risk of bias tool

We assessed the risk of bias for each study using the criteria of the *Cochrane Handbook for Systematic Reviews of Interventions* [24]. The criteria included in the risk of bias tool are: random sequence generation (selection bias), allocation concealment (selection bias), blinding of participants and personnel (performance bias), blinding of outcome assessment (detection bias), incomplete outcome data (attrition bias), and selective reporting (reporting bias).

We identified other possible sources of bias specific to the literature that were not covered elsewhere in the tool. Thus, we added 4 criteria: i) blinding of participants to apparatus display, ii) blinding of therapist to apparatus display, iii) blinding of the data extraction / cleaning process, and iv) blinding of the statistician.

The reasons for these added observations are that several outcome variables used to assess autonomic mediated physiology are captured directly on computer and can be displayed on a screen as a graph during the experiment. Thus, blinding of both therapist and subject to apparatus display is an important issue, as purposeful or subconscious data management or interpretation may affect the results. Indeed, if there were any feedback, the subjects could be disturbed or influenced by the screen with data variations and the therapist could modify his intervention to obtain higher or lower values. Further, the data extraction (selection of relevant blocks from the recording) and especially the cleaning process of the raw data should also be blinded, as the final results depend on the choice of data and a proper cleaning process (e.g. removal of artifacts, extrasystoles). Additionally, the statistician should also be blinded to avoid bias in the choice and interpretation of analytical methods and post hoc tests [25].

#### Use of the risk of bias tool

Two reviewing authors independently assessed the risk of bias for each study. We used a consensus method to resolve any disagreement and a third author would be consulted if the disagreement persisted. Each criterion was judged as low risk, unclear risk, or high risk of bias following the criteria for judgement available in the *Cochrane Handbook for Systematic Reviews of Interventions* [24], C*hapter 8, Table 8.5.d.* The blinding of participants (performance bias) was judged as low risk, if it was tested with a post-trial questionnaire (or equivalent) and found acceptable. When there was insufficient information, criteria were judged as unclear risk. The results of the risk of bias assessment for each item, and detailed information with support for judgment are available in Additional file 2.

#### Summary assessment of the risk of bias for each study

For the summary assessment we mainly based our judgment on domains that we consider critical in this type a research. These domains are blinding of participants (performance bias), blinding of outcome assessment (detection bias), blinding of both participants and therapist to apparatus display, blinding of the data extraction / cleaning process, and blinding of the statistician. Items dealing with ‘blinding’ are important, as autonomic mediated physiology is likely to be influenced by feelings resulting from knowledge of the intervention; thus blinding of participants and outcome assessment are critical. In addition, blinding of the therapist, data extraction / cleaning process, and data analysis are also important, as research team members participating in these processes can conscientiously or subconsciously bias the data. Unclear or high risks of bias in these domains are likely to result in ‘positive’ outcomes in favor of the tested intervention, as the effect would probably be larger if the sham procedure is ineffective, and because the research team implicating in data collection and management are also likely to promote ‘positive’ outcomes.

Other domains of bias, i.e. randomization process, attrition, and selective reporting were taken into account in the assessment only if they were judged as ‘high’ risk of bias, as we considered minor methodological omissions that lead to ‘unclear’ risk of bias to be of little importance in this type of experimental studies.

### 2/ Technical quality of the studies

#### Rationale for technical quality check-list

Inaccurate data are not only caused by willful or subconscious influences or interpretations by study subjects or members of the research team (so-called ‘bias’ or ‘systematic errors’). In experimental studies, inaccuracies can also be brought about by carelessness in relation to the technical aspects. Several important technical points to consider (different from risk of bias) have previously been recommended in order to obtain valid data when conducting experimental studies on autonomic mediated physiology such as SC [26], HRV [27], and SBF [28]. Therefore, determining the effect of JMT on autonomic mediated physiology requires careful control over several factors relating to the stabilizing period before baseline recordings [27, 28], to the environment (e.g. temperature, noise) [26, 28], intake of stimulants (e.g. caffeine, tobacco) [27, 28], recordings (e.g. sampling rate) [26, 27, 29], and processing of data (e.g. removal of artefacts) [27, 28]. It is also important to consider whether recordings were carried out by an experienced person and whether they are reliable or reproducible as factors such as preparation of the skin and placement of the electrodes may lead to measurements errors [26, 28], e.g. poor reproducibility [28]. It is also important to consider, when interpreting results, if the study sample was sufficiently large (e.g. based on a power calculation) [28]. However, there is no one single well-established tool to assess this domain because different types of experimental studies have different technical requirements. Therefore, study-specific check-lists must be created to deal with this important aspect to judge the veracity of data in addition to the usual risk of bias assessment.

#### Development of the technical quality assessment check-list

For the purpose of this review, a technical quality assessment check-list was therefore developed in collaboration with an expert in the area (DH), aided by an engineer specialized in technical measurements of physiological outcomes, and also taking into consideration previous studies and own experience of this type of studies (MP). The tool was thereafter tested on a number of articles by three authors (MP, CLY, DH), adjusted after further discussions, and taken into use. As there was no disagreement between the authors on these items during the data extraction, we concluded that it was user-friendly.

#### Contents of technical quality check-list

The items in the technical quality check-list (Additional file 3**)** have been described and explained below.

**Item 1** reports if the JMT was performed by a qualified person.

**Item 2** reports if the JMT and sham procedures were sufficiently described in such a way to be possible to be reproduced by others.

**Item 3** reports if the main outcome measurement was reported as reliable or reproducible either by referring to previous work or through testing of own data.

**Item 4** reports if the acquisition of data was well performed relating to five aspects:

- Attempts should have been made to limit or control factors that can affect autonomic mediated physiology (e.g. intake of food, caffeine, tobacco, alcohol, temperature, noise, hour of the day).
- The measurement procedure should be described to enable replication of the study and judge possible sources of bias.
- There should be a minimum rest period before the measurement to stabilize autonomic mediated physiology. The duration of the stabilizing period before the beginning of the baseline measurement was set at 5 min. We considered this duration acceptable without being too restrictive. Since we did not find any guidelines, this value was based on recommendations for blood pressure measurements [30] and used by *Laborde* et al. [27] in their recommendations for HRV. However, higher values may be better, as stated by *Zegarra-Parodi* et al. [28] for skin blood flow.
- The measurements should be performed by an experienced person.
- The sampling rate should be adequate. This point is crucial as most of the measurements of the ANS result from the continuous acquisition of a physiological signal. Therefore, if the sampling rate is too low, information is definitely lost, and the original signal will not be represented correctly and the data inoperable. We considered 1000 Hz to be an adequate sampling rate in a research setting for HRV [29] but lower sampling rates are also proposed (e.g. 125 Hz, 500 Hz) [27], and 20 Hz as a minimum for SC [26], skin temperature, and skin blood flow.

**Item 5** reports if the data cleaning process was described, as the validity of the outcome variable depends on a proper cleaning process (e.g. removing artefacts).

**Item 6** refers to whether the number of subjects was based on a power calculation performed on the primary autonomic-related outcome variable, so that a lack of significant results could not be attributed to a lack of power.

#### Additional item *(not included in the technical quality score)*

As this review relates to sham-controlled studies, we also reported in Additional file 3 an item dealing with the *mechanical* profile of the sham procedures. In fact, any stress (e.g. mechanical stress) can trigger ANS responses [31]. Therefore, in a context of autonomic measurements, it is relevant to discuss the possible impact of the *mechanical* profile of the different sham procedures on the results (e.g. inert sham versus sham adopting a mechanical profile similar to the JMT). This item was used to give rise to a discussion and not as a criterion to judge the quality of studies.

#### Use of the technical quality check-list

The technical quality assessment was performed by two independent researchers. If needed, a third investigator would be consulted to reach agreement. Points were given for these items as shown in Additional file 3. One point was given to each item or sub-item. The final score was reported as a percentage. The studies were arbitrarily considered as having a low technical quality, if their score was below 50%. This level was chosen because no ‘official’ cut point exists and because it is conservative, i.e. as the technical check-list is based on fundamental technical points recommended in previous literature to assess autonomic mediated physiology, we consider that it is reasonable to have a doubt about the results, when a study respects less than half of these basic items. The technical quality was further used in the GRADE approach to assess the certainty of evidence. The technical quality assessment (with details) and the score are transparently reported in Additional file 3.

### 3/ Assessment of the certainty of evidence

#### Cochrane GRADE approach

The GRADE approach [24] contains four levels to judge the certainty of evidence for each variable under scrutiny based on the whole field of research: ‘high’ (further research is very unlikely to change our confidence in the estimate of effect), ‘moderate’ (further research is likely to have an important impact on our confidence in the estimate of effect and may change the estimate), ‘low’ (further research is very likely to have an important impact on our confidence in the estimate of effect and is likely to change the estimate), ‘very low’ (we are very uncertain about the estimate).

#### Use of the GRADE approach in this review

We assessed the certainty of evidence for changes in autonomic activity based on changes in autonomic mediated physiology (markers of autonomic activity) for all comparisons. The certainty of evidence started at ‘high’ for randomized controlled trials and was downgraded by one level (for serious concerns) or two levels (for very serious concerns) based on several domains detailed below.

We downgraded the certainty of evidence for:

- *Limitation in study design (risk of bias)*. Please note that if the studies were judged as having unclear or high risk of bias, based on domains dealing with ‘blinding’ (see above), we downgraded the certainty of evidence only if an overall ‘effect’ was reported for the outcome variable, as risk of bias in these domains would be in favor of an effect.
- *Inconsistency*, if similar studies reported statistically significant effects in opposite directions or if they reported ‘effect’ and ‘no effect’.
- *Indirectness,* if the markers do not provide some quantitative measures of autonomic activity, e.g. ‘mean’ heart rate or ‘mean’ blood pressure was used instead of analyzing heart rate variability or blood pressure variability. We downgraded for indirectness if the markers are not well accepted for assessing autonomic mediated physiology, e.g. *skin temperature* and *skin blood flow* (see Discussion) or *respiratory rate*.
- *Possible imprecision*, if our final conclusions were based on less than 5 studies for each outcome variable. We chose this threshold to be conservative considering that our conclusions per outcome were, generally, based on a low number of studies. However, we could not follow the Cochrane guidelines for this domain [32], which are designed for clinical research (e.g. rating down for imprecision when the sample size is inferior to 400 subjects), as we dealt mainly with experimental research, (i.e. controlled studies with small sample sizes).
- *Publication bias,* please see *Cochrane Handbook* [24].
- *Technical issues*, if the technical quality score was low (< 50%) in one or several studies for a particular outcome and judged to be a limitation. We added this domain to the classical GRADE approach to fit with this particular field of research. We clearly reported in the results section where this domain was used for downgrading the certainty of evidence.

### Data analysis and synthesis

#### Data analysis

Studies were sorted according to the JMT employed and the year of publication. Between-group / intervention differences, i.e. the difference in outcome for JMT vs Sham with a statistical test, were selected as the main result for each outcome variable. We reported an ‘effect’ when there was a statistically significant difference between the JMT and the Sham. The results for every study and every outcome variable are reported in the result table (Additional file 4).

When data were available from at least 2 studies, and if the nature of the outcome and other key aspects of studies were judged to be similar enough, we performed a meta-analysis based on the mean difference between the JMT and the sham, using the *RevMan 5.3 software* distributed by *Cochrane* [33]. We used the inverse variance method with a fixed-effects model or with a random-effects model if the I^2^ statistic was superior to 50% and the heterogeneity could not be explained [24].

Cross-over studies often did not provide all relevant statistical information for a meta-analysis based on between-groups differences. However, if they provided relevant standard deviations for each intervention (JMT and sham), we used two approaches to perform the analysis. First, we analyzed them as parallel group trials (this method could give rise to unit-of-analysis error, but it is a conservative approach, *Cochrane handbook, chapter 16.4.5* [24]. Second, we also calculated the standard error of the difference by hypothesizing different correlation coefficients, as proposed in the Cochrane *Handbook, chapter 16.4.6.1 and 16.4.6.3* [24]*.* The different correlation coefficients were 0.1 (low positive correlation), 0.5 (medium positive correlation) and 0.9 (high positive correlation). Finally, we conducted a sensitivity analysis to investigate the effect of the different approaches (available from the authors on request).

It there were enough data (at least 2 studies per subgroup), we would conduct subgroup analyses to investigate if the estimate of effect differed between studies on healthy and symptomatic subjects.

Unless the data could be pooled in meta-analysis, the studies, which did not report between-groups difference although finding significant within-group difference, were not included in the synthesis of the results, as their results can be misleading [34].

#### Data synthesis

We undertook a structured synthesis and reported the findings narratively with the certainty of evidence (GRADE) along with justifications for decisions to downgrade the certainty of evidence following the recommendations described in the Cochrane Handbook. To make the results easier to read, we have provided a summary of findings table (key information), which recapitulates the narrative synthesis (Table 2).

## Results

### Study characteristics

**Fig. 1:**
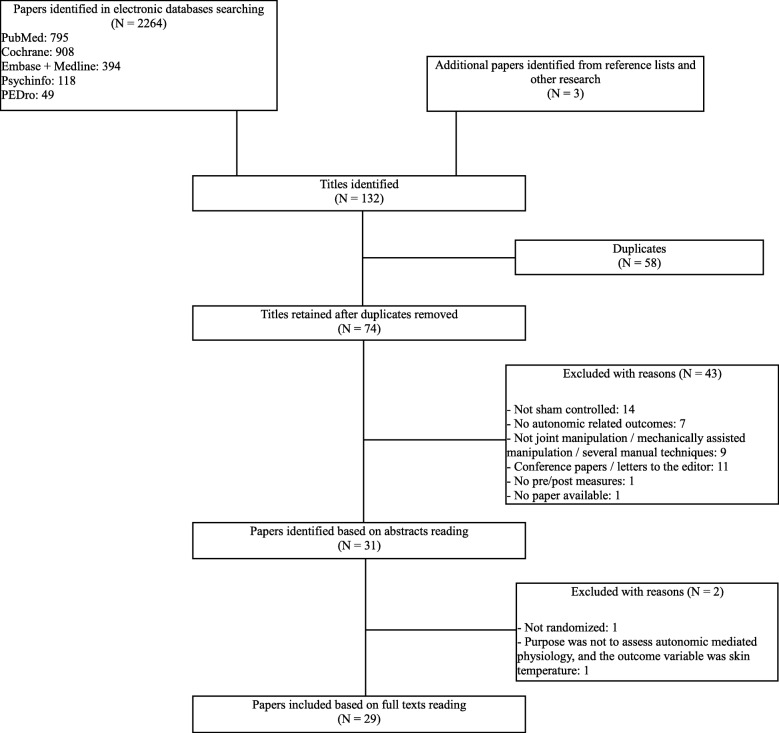
(attached file): Flow chart of the search and selection process

Of the 2267 screened studies we first retained 29 suitable studies, all in English (Fig. 1). Of these, 16 deal with mobilizations (oscillatory technique) [35–50], 1 with an atypical mobilization technique [51], 5 with sustained natural apophyseal glides (SNAGs) / mobilization with movement [52–56], and 7 with high velocity low amplitude manipulation [57–63]. The experimental interventions were undertaken in various parts of the spine but also in the extremities in two studies [38, 52] and the effects were reported in relation to skin conductance (*N* = 15), heart rate (*N* = 11), skin temperature (*N* = 11), blood pressure (*N* = 7), HRV (*N* = 5), respiratory rate (*N* = 3), skin blood flow (*N* = 3), biochemical markers (*N* = 2), pupil diameter (*N* = 1), and oxy-hemoglobin concentration (*N* = 1). Outcomes were generally measured during the intervention, often directly afterwards, but sometimes also longer after the experimental intervention. Studies would often report on more than one outcome variable, which explains why the total number of outcome variables is larger than the number of studies. Twenty-three studies dealt with healthy subjects whereas 6 used symptomatic study subjects. This information and other items are found in Additional file 1.

Two studies [57, 58] did not test for between-group differences but available data could be included in various meta-analyses to compare the intervention to a sham.

### Risk of bias in included studies

Overall, we judged 14/16 studies dealing with mobilizations (oscillatory technique), 5/5 studies dealing with SNAGs / mobilization with movement, and 5/7 studies dealing with HVLA manipulation as having unclear risk of bias. This judgment was based on the unclear risk present in categories to which we paid most attention, such as blinding of participants, blinding of the data extraction / cleaning process, and blinding of the statistician. We judged two studies [47, 50] assessing the effects of mobilizations with oscillatory movements as having probably a low risk of bias. Three studies, two dealing with HVLA manipulation [57, 58], and one dealing with an atypical technique [51] were judged as having high risk of bias given that the subjects were not well blinded to the intervention. Unclear or high risk of bias in these categories would be in favor of an effect of the JMT compared to sham, thus we would use this risk for downgrading the certainty of the evidence only if a statistically significant effect was reported.

**Fig. 2:**
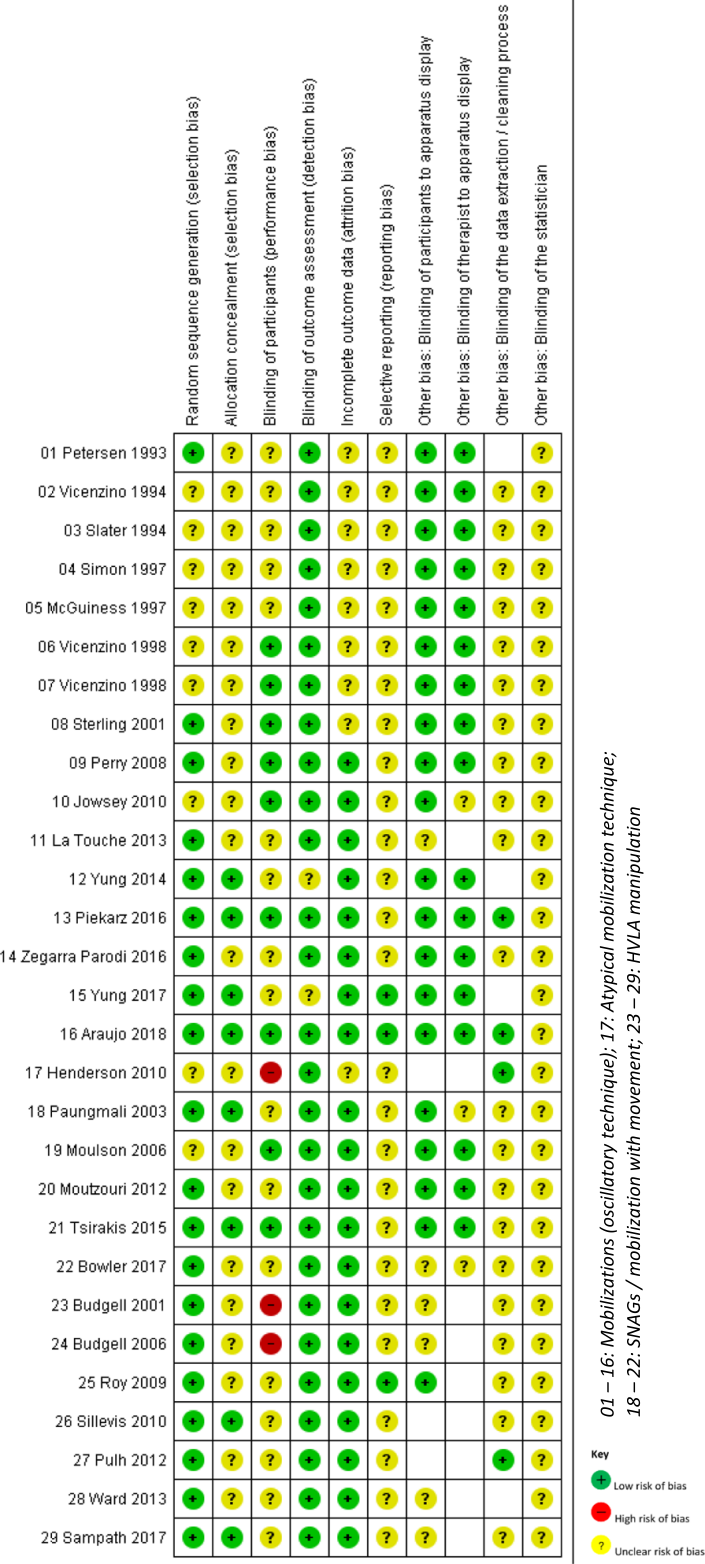
(attached file): Risk of bias summary: review authors’ judgements about each risk of bias item

The results of the risk of bias assessment for each study are summarized in Fig. 2. Support of judgment for each item is provided in Additional file 2.

### Overall technical quality

We judged the technical quality of the included studies as being acceptable in 25/29 studies (score ≥ 50%). However, 4/29 studies, 2 dealing with mobilizations (oscillatory technique) [40, 41] and 2 dealing with HVLA manipulation [57, 59] were judged as probably being technically deficient, since they had a low technical score (< 50%). Overall, the items that were often missing or not fulfilled correctly were the reliability or reproducibility of the outcome variables, the data cleaning process, and the experience of the assessor. For a summary of findings and details, please see Additional file 3.

### Sham procedure

The vast majority of the included studies (25/29) defined the sham as an ‘inactive’ manual contact (without movement) over the area of intervention, 2/29 studies [57, 58] used a sham, which was mechanically similar to the true intervention without involving joints and surrounding tissues. One study [48] used a sham similar to the true intervention with less pressure, and one study [40] did not describe the sham procedure (Additional file 3). Twelve studies [40–44, 47, 50, 51, 53, 55, 57, 58] assessed, if the participants were well blinded to the interventions delivered, using a post-trial questionnaire, and thus if the sham procedure was likely to produce the same expectations as with the true intervention. In 9/12 studies [40–44, 47, 50, 53, 55], all on various types of mobilizations, blinding had been established to have been successful (Fig. 2, Additional file 2).

### Effects of interventions

The effects for each joint manipulative technique compared to sham are reported narratively below for each outcome variable. Detailed results for each study are available in Additional file 4. A summary of the narrative synthesis (i.e. key information) is provided in the Table 2. The results are reported with our assessment of the certainty of evidence. The GRADE assessment started with a baseline rating of high-certainty because we included only randomized controlled trials. The subsequent reasons for downgrading the certainty of evidence are reported in the Table 3.

**Table 2:** Summary of findings

Outcome	Results	Summary with the certainty of evidence (GRADE)
Mobilizations (oscillatory technique) compared to sham		
Skin conductance (1) (sudomotor activity)	Mobilizations increased SC compared to sham in 10/10 studies. Mobilizations produced mean percentage changes of SC ‘integral measurement’ that were 13.75 and 9.34% greater than with the sham respectively for change from baseline to intervention period and change from baseline to post intervention period (3 studies in meta-analysis).	Mobilizations probably produce a bilateral increase in skin sympathetic nerve activity during both the intervention and immediate post intervention periods regardless of the area treated (moderate-certainty evidence).
Skin temperature (1) (vasomotor activity)	Mobilizations had no effect on skin temperature in 5/8 studies which could not be included in meta-analysis.	We are uncertain whether mobilizations have no acute effect on skin sympathetic nerve activity (very low-certainty evidence).
Skin blood flow (1) (vasomotor activity)	Mobilizations had no effect on skin blood flow in 1/2 studies, while the other study found effects in opposite directions (both increase and decrease). Studies could not be included in meta-analysis.	*Please note that we based our conclusions on skin conductance (higher certainty of evidence). See narrative synthesis for details.*
Heart rate (2)	Mobilizations had, generally, no effect on heart rate. There was no effect during both the intervention (2 studies included in meta-analysis) and post intervention periods (3 studies included in meta-analysis). Another study also reported no effect. However, 2 studies found a significant increase during the intervention period, and another study reported a significant increase during the post intervention period.	Mobilizations may have no acute effect on cardiovascular autonomic activity (very low- to low-certainty evidence).
Blood pressure (2)	Mobilizations had, generally, no effect on blood pressure. There was no effect during the intervention and post intervention periods (2 studies included in meta-analysis). Another study also reported no effect. However, 2 studies found a significant increase compared to sham.	
Heart rate variability (2)	Mobilizations had no effect on HRV in 1/1 study.	
Respiratory rate (3)	Mobilizations increased respiratory rate compared to sham in 3/3 studies which could not be included in meta-analysis.	We are uncertain whether mobilizations increase respiratory rate via an increase in sympathetic activity (very low-certainty evidence).
Atypical mobilization technique compared to sham		
Salivary alpha amylase activity (4)	An atypical mobilization technique decreased the salivary alpha amylase activity compared to sham in 1/1 study.	We are uncertain whether an atypical mobilization technique decreases the salivary glands sympathetic nerve activity (very low-certainty evidence).
(1) Markers used to assess skin sympathetic nerve activity; (2) Markers used to assess cardiac / cardiovascular autonomic activity; (3) Marker used to assess ‘non-specific’ sympathetic arousal; (4) Marker used to assess the salivary glands sympathetic nerve activity		
Outcome	Results	Summary with the certainty of evidence (GRADE)
SNAGs / Mobilization with movement compared to sham		
Skin conductance (1) (sudomotor activity)	Spinal SNAGs had no effect on SC in 3/4 studies. Two studies could be included in meta-analysis, the results showed no statistically significant difference between the technique and the sham for both intervention and post intervention periods.	Spinal SNAGs may have no acute effect on skin sympathetic nerve activity (low-certainty evidence).
	A peripheral (elbow) mobilization with movement increased SC compared to sham in 1/1 study.	We are uncertain whether peripheral ‘mobilization with movement’ techniques increase skin sympathetic nerve activity (very low-certainty evidence).
Skin temperature (1) (vasomotor activity)	Spinal SNAGs had no effect on skin temperature in 2/2 studies which could not be included in meta-analysis.	We are uncertain whether spinal SNAGs have no acute effect on skin sympathetic nerve activity (very low-certainty evidence). *Please note that we based our conclusions on skin conductance (higher certainty of evidence).*
Skin blood flow (1) (vasomotor activity)	A peripheral (elbow) mobilization with movement increased or decreased skin temperature and skin blood flow compared to sham in 1/1 study according to the measurement area.	We are uncertain whether peripheral ‘mobilization with movement’ techniques modulate skin sympathetic nerve activity (very low-certainty evidence).
Heart rate (2)	A peripheral (elbow) mobilization with movement increased heart rate and blood pressure compared to sham in 1/1 study.	We are uncertain whether peripheral ‘mobilization with movement’ techniques modulate cardiovascular autonomic activity (very low-certainty evidence).
Blood pressure (2)		
(1) Markers used to assess skin sympathetic nerve activity; (2) Markers used to assess cardiac / cardiovascular autonomic activity		
Outcome	Results	Summary with the certainty of evidence (GRADE)
Spinal manipulation (HVLA technique) compared to sham		
Heart rate variability (1)	Spinal manipulation had no effect on - various HRV components (results from respectively 3/3 and 4/4 studies included in meta-analysis)	Spinal manipulation may have no acute effect on cardiovascular autonomic activity (very low- to low-certainty evidence). *Please note that we downgraded the certainty of evidence by one level for technical issues*.
Heart rate (1)	- Heart rate (results from 3/3 studies included in meta-analysis)	
Blood pressure (1)	- Blood pressure in 1/1 study	
Pupil diameter (2) (pupillometry)	Spinal manipulation had no effect on pupil diameter in 1/1 study.	Spinal manipulation may have no acute effect on the autonomic control of the pupil (low-certainty evidence).
Plasma concentrations of epinephrine and norepinephrine (3)	Spinal manipulation had no effect on the plasma concentrations of epinephrine and norepinephrine in 1/1 study.	Spinal manipulation may have no acute effect on the sympathoadrenal system activity (low-certainty evidence).
Oxy-hemoglobin concentration (4)	Spinal manipulation had no effect on the oxy-hemoglobin concentration measured on the gastrocnemius in 1/1 study.	We are uncertain whether spinal manipulation has no acute effect on muscle sympathetic nerve activity (very low-certainty evidence).
(1) Markers used to assess cardiac / cardiovascular autonomic activity; (2) Marker used to assess the autonomic control of the pupil; (3) Markers used to assess the sympathoadrenal system activity; (4) Marker used to assess muscle sympathetic nerve activity		

Please note that in the included studies *skin conductance, skin temperature, and skin blood flow* were used as markers of the skin sympathetic nerve activity. *Heart rate, heart rate variability, and blood pressure* were used as markers of the cardiac / cardiovascular autonomic activity. *Respiratory rate* was used as a ‘non-specific’ marker of sympathetic arousal. *Alpha amylase activity* was used as a marker of the sympathetic activity in the salivary glands. *Pupil diameter* was used as a marker of the autonomic control of the pupil*. Plasma concentrations of epinephrine and norepinephrine* were used as markers of the sympathoadrenal system activity*.* Finally, *oxy-hemoglobin* concentration was used as a marker of muscle sympathetic nerve activity*.*

### 1/ Mobilizations (oscillatory technique) versus sham

#### Outcome: Skin conductance

Moderate-certainty evidence (Table 3 A) suggests that mobilizations (oscillatory technique using various frequencies) probably produce a bilateral increase in skin sympathetic nerve activity during both the intervention and the immediate post intervention periods, as shown by the statistically significant increase in SC compared to sham, reported in 10/10 studies [35–38, 41–45, 47]. The effect was clearly reported to occur during the intervention period in 6/7 studies and during the post intervention period in 3/5 studies. In addition, 4/6 studies found the effect to be bilateral. Mobilizations were applied mainly on the different regions of the spine (cervical, thoracic or lumbar); one study testing a peripheral (shoulder) technique. Three studies used symptomatic subjects.

The data from 3 studies [43, 44, 47] could be pooled in a meta-analysis. Spinal mobilizations produced a mean percentage change from baseline to intervention period in SC ‘integral measurement’ that was 13.75% greater than with the sham (mean difference 13.75, 95% CI 1.36 to 26.14, I^2^ = 51%, random effect, *p* = 0.03; 3 studies, 96 subjects) and 9.34% greater than with the sham from baseline to post intervention period (mean difference 9.34, 95% CI 2.85 to 15.83, I^2^ = 0%, *p* = 0.005; 3 studies, 96 subjects).

#### Outcome: Skin temperature

Very low-certainty evidence (Table 3 B, C) suggests that mobilizations have no acute effect on skin sympathetic nerve activity, as there was no effect on skin temperature during the intervention or during the immediate post intervention period in 5/8 studies [35–37, 45, 48]. Studies could not be pooled in meta-analysis as relevant data were not available.

#### Outcome: Skin blood flow

Very low-certainty evidence (Table 3 D, E, P) suggests that mobilizations may modulate (increase and decrease) or may have no effect on skin sympathetic nerve activity, as there was no effect on skin blood flow in 1/2 studies [48], while the other study [41], found effects in opposite directions (both increase and decrease). Studies could not be pooled in meta-analysis from lack of relevant data.

Please note that we downgraded the certainty of evidence for indirectness (Table 3 C, E) for skin temperature and skin blood flow, as there is evidence indicating that they are not good markers of skin sympathetic nerve activity (please see Discussion), whereas skin conductance is acceptable. Given that there are contradictory results for this comparison (mobilizations versus sham) between, on one side, skin conductance, and on the other side skin temperature and skin blood flow, we based our conclusion on skin conductance (higher certainty of evidence).

#### Outcome: Heart rate

Very low-certainty evidence (Table 3 F, G, P) suggests that mobilizations have no acute effect on cardiovascular autonomic activity, as there was no effect on heart rate in beats per minute (bpm) during the intervention period in 2 pooled studies [46, 49] (mean difference − 0.83 bpm, 95% CI -5.47 to 3.81, I^2^ = 0%, *p* = 0.73; 83 subjects) and during the immediate post intervention period in 3 pooled studies [46, 49, 50] (mean difference − 1.23 bpm, 95% CI -4.47 to 2.02, I^2^ = 0%, *p* = 0.46, 121 subjects). A fourth study [48] also reported no effect during the immediate post intervention period. However, two studies [39, 40] found a statistically significant increase in heart rate from baseline to intervention period, and another study [45] reported a statistically significant increase during the immediate post intervention period.

#### Outcome: Blood pressure

Very low-certainty evidence (Table 3 H, I, P) suggests that mobilizations have no acute effect on cardiovascular autonomic activity, as there was no effect on systolic blood pressure during the intervention period in 2 pooled studies [46, 49] (mean difference − 2.02 mmHg, 95% CI -6.96 to 2.92, I^2^ = 31%, *p* = 0.42, 83 subjects) and during the immediate post intervention period in the same pooled studies (mean difference − 1.02 mmHg, 95% CI -5.77 to 3.72, I^2^ = 0%, *p* = 0.67, 83 subjects). There was also no effect on diastolic blood pressure, during the intervention period (mean difference − 0.07 mmHg, 95% CI -3.09 to 2.94, I^2^ = 0%, *p* = 0.96, 83 subjects) and during the immediate post intervention period (mean difference 0.32 mmHg, 95% CI -2.49 to 3.14, I^2^ = 0%, *p* = 0.82, 83 subjects). Another study [48] also reported no statistically significant difference in mean arterial blood pressure during the immediate post intervention period. However, 2 studies [39, 40] found a statistically significant increase in systolic blood pressure (percentage change from baseline to intervention period) compared to sham.

#### Outcome: Heart rate variability

Low-certainty evidence (Table 3 P) suggests that mobilizations may have no acute effect on cardiac autonomic activity, as there was no effect on HRV immediately after the intervention in 1/1 study [50].

#### Outcome: Respiratory rate

Very low-certainty evidence (Table 3 J, K, P, Q) suggests that mobilizations produce a statistically significant increase in respiratory rate compared to sham in 3/3 studies [39, 40, 45] via an increase in sympathetic activity. The effects were found to occur during or immediately after the intervention. Studies could not be pooled in meta-analysis as relevant data were not available.

Please note that for *respiratory rate* we used a modified GRADE approach, as we downgraded the certainty of evidence by one level for technical issues (Table 3 Q*)*.

### 2/ Atypical mobilization technique versus sham

#### Outcome: Alpha amylase activity

Very low-certainty evidence (Table 3 L, P) suggests that an atypical mobilization technique produces an acute decrease of the sympathetic activity in the salivary glands, as there was a statistically significant decrease in salivary alpha amylase compared to a sham within 10 min after the intervention in 1/1 study [51].

### 3/ Spinal SNAGs / mobilization with movement versus sham

#### Outcome: Skin conductance

Low-certainty evidence (Table 3 M, P) suggests that spinal SNAGs may have no acute effect on skin sympathetic nerve activity, as there was no effect on SC during the intervention period in 4/4 studies [53–56] and during the immediate post intervention period in 3/4 studies [54–56].

The data from two studies [54, 55] could be pooled in a meta-analysis. There was no effect on SC ‘integral measurement’ for change from baseline to intervention period (mean difference 4.62, CI 95% -2.31 to 11.55, I^2^ = 0%, *p* = 0.19, 2 studies, 60 subjects) and for change from baseline to post intervention period (mean difference 3.99, CI 95% -3.47 to 11.44, I^2^ = 0%, *p* = 0.29, 2 studies, 60 subjects).

#### Outcome: Skin temperature

Very low-certainty evidence (Table 3 C, P) suggests that spinal SNAGs have no acute effect on skin sympathetic nerve activity, as there was no effect on skin temperature both during the intervention and the immediate post intervention periods in 2/2 studies [53, 56]. Studies could not be pooled in meta-analysis, as relevant data were unavailable.

### 3.1/ Peripheral SNAGs / mobilization with movement versus sham

#### Outcome: Skin conductance

Very low-certainty evidence (Table 3 N, P) suggests that peripheral mobilization with movement techniques increase skin sympathetic nerve activity as there was a statistically significant increase in SC compared to sham during the intervention period in 1/1 study [52].

#### Outcome: Skin temperature, skin blood flow

Very low-certainty evidence (Table 3 C, E, N, P) suggests that peripheral mobilization with movement techniques modulate skin sympathetic nerve activity, as there was a statistically significant increase or decrease in skin temperature and skin blood flow compared to sham during the intervention period in 1/1 study [52].

#### Outcome: Heart rate, blood pressure

Very low-certainty evidence (Table 3 G, I, N, P) suggests that peripheral mobilization with movement techniques modulate cardiovascular autonomic activity, as there was a statistically significant increase in heart rate and blood pressure compared to sham (from baseline to the immediate post intervention period) in 1/1 study [52].

### 4/ HVLA manipulation versus sham

#### Outcome: Heart rate variability

Low-certainty evidence (Table 3 P, Q) suggests that spinal manipulation may have no acute effect on cardiac autonomic activity, as there was no effect on various HRV components immediately after the intervention. There was no effect on the spectral power of the normalized HF component (mean difference 1.16, 95% CI -4.86 to 7.18, I^2^ = 0%, *p* = 0.71, 3 studies [57–59]), no effect on the spectral power of the normalized LF component (mean difference 2.84, 95% CI -3.47 to 9.14, I^2^ = 0%, *p* = 0.38, 3 studies [57–59]), and no effect on the LF / HF ratio (mean Difference − 0.06, 95% CI -0.34 to 0.22, I^2^ = 0%, *p* = 0.67, 4 studies [57–59, 63]). *Sampath* et al. [63] also reported no effect on the LF / HF ratio 30 min after the intervention.

We conducted a sensitivity analysis, as two studies used a cross-over design (please see Method). In all cases, the test for overall effect was not statistically significant. Results are reported with the conservative approach considering cross-over trials as parallel group trials.

#### Outcome: Heart rate

Very low-certainty evidence (Table 3 G, P, Q) suggests that spinal manipulation has no acute effect on cardiovascular autonomic activity, as there was no effect on heart rate immediately after the intervention (mean difference − 1.67 bpm, 95% CI -5.33 to 1.98, I^2^ = 1%, *p* = 0.37, 3 studies [57, 58, 62]). *Ward* et al. [62] also reported no effect on heart rate 10 min and 24 h after the intervention.

We conducted the same sensitivity analysis as stated above. In all cases, the test for overall effect was not statistically significative. Results are reported with the conservative approach considering cross-over trials as parallel group trials.

Please note that for *heart rate variability* and *heart rate* we used a modified GRADE approach, as we downgraded the certainty of evidence by one level for technical issues (Table 3 Q).

#### Outcome: Blood pressure

Very low-certainty evidence (Table 3 I, P) suggests that spinal manipulation has no acute effect on cardiovascular autonomic activity as there was no effect on blood pressure immediately, 10 min, and 24 h after the intervention in 1/1 study [62].

#### Outcome: Pupil diameter

Low-certainty evidence (Table 3 P) suggests that spinal manipulation may have no acute effect on the autonomic control of the pupil, as there was no effect on pupil diameter within 5 min after the intervention. in 1/1 study [60].

#### Outcome: Plasma concentrations of epinephrine and norepinephrine

Low-certainty evidence (Table 3 P) suggests that spinal manipulation may have no acute effect on the sympathoadrenal system activity, as there was no effect on the plasma concentrations of epinephrine and norepinephrine immediately and 15 min after the intervention in 1/1 study [61].

#### Outcome: Oxy-hemoglobin concentration

Very low-certainty evidence (Table 3 O, P) suggests that spinal manipulation has no acute effect on muscle sympathetic nerve activity, as there was no effect on the oxy-hemoglobin concentration measured on a calf muscle immediately, 5 min, and 30 min after the intervention in 1/1 study [63].

**Table 3:** Reasons for downgrading the certainty of evidence

		Downgraded by
A	Risk of bias, 9/10 studies were judged as having unclear risk of bias (unclear risk concerning the blinding of the participants, blinding of the data extraction / cleaning process and blinding of the statistician). Some inconsistency, as 2/6 studies found the effect not to be bilateral and 2/5 not to be present during the post intervention period.	1 level
B	Inconsistency, as 3/8 studies reported a statistically significant effect, the others not.	1 level
C	Indirectness, as there is evidence indicating that skin temperature is not a good marker of skin sympathetic nerve activity (as explained in the Discussion).	2 levels
D	Inconsistency, as studies found both effect and non-effect.	1 level
E	Indirectness, as there is evidence indicating that skin blood flow is not a good marker of skin sympathetic nerve activity (as explained in the Discussion).	2 levels
F	Inconsistency, as 3 studies which could not be pooled in the meta-analysis reported a statistically significant effect.	1 level
G	Indirectness, heart rate variability is a better outcome to assess cardiac autonomic activity (as explained in the Method).	1 level
H	Inconsistency, as 2 studies which could not be pooled in the meta-analysis reported a statistically significant effect.	1 level
I	Indirectness, blood pressure variability is a better outcome to assess cardiovascular autonomic activity (as explained in the Method).	1 level
J	Risk of bias, 3/3 studies were judged as having unclear risk of bias (unclear risk concerning the blinding of the participants, blinding of the data extraction and blinding of the statistician).	1 level
K	Indirectness, respiratory rate seems not to be a well-accepted outcome to assess autonomic activity.	1 level
L	Risk of bias, the study was judged as having high risk of bias (lack of blinding of the participants).	2 levels
M	Inconsistency, as one study which could not be pooled in the meta-analysis reported a statistically significant effect.	1 level
N	Risk of bias, the study was judged as having unclear risk of bias (unclear risk concerning the blinding of the participants, blinding of the data extraction and blinding of the statistician).	1 level
O	Possible indirectness, oxy-hemoglobin concentration is an indirect measure of muscle blood flow.	1 level
P	Imprecision, one study (downgraded by two levels); two to four studies (downgraded by one level).	1 or 2 level(s)
Q	Technical issues, as 1/3 study for *respiratory rate,* 2/3 or 2/4 studies for *Heart rate variability,* and 1/3 study for *Heart rate* had a low technical score.	1 level

## Discussion

### Brief summary of findings

In summary, we included 29 randomized sham-controlled trials dealing with several joint manipulative techniques in this systematic review and our evaluation of the literature suggests that, as compared to sham interventions:

- *Mobilizations (oscillatory technique)* probably produce an immediate and short-term, bilateral increase in skin sympathetic nerve activity (increased sudomotor activity), regardless of the area treated (moderate-certainty evidence). This effect was measured for only a maximum of 5–10 min after intervention, for which reason its duration is unknown. It is uncertain whether the sympathetic arousal also explains an increase in respiratory rate (very low-certainty evidence). This technique may have no acute effect on cardiovascular autonomic activity (very low- to low-certainty evidence).
- *Spinal SNAGs / mobilization with movement* may have no acute effect on skin sympathetic nerve activity (very low- to low-certainty evidence). We are uncertain whether *peripheral ‘mobilization with movement’* techniques increase skin sympathetic nerve activity or modulate cardiovascular autonomic activity, as the certainty of the evidence was assessed as very low.
- S*pinal manipulation (HVLA technique)* may have no acute effect on cardiovascular autonomic activity and on various other markers of autonomic activity (very low- to low-certainty evidence).

The certainty of evidence (using a modified GRADE approach) was often assessed as very low or low, thus further research is likely to change these conclusions. The certainty of evidence was mainly downgraded because of inconsistency, indirectness, imprecision, risk of bias, and to a lesser extent, for insufficient technical quality.

### Agreements and disagreements with other studies or review

Regarding mobilizations (oscillatory technique), our findings are in agreement with the interpretation provided in two previous reviews concerning the increase in skin sympathetic nerve activity (increase in *skin conductance,* i.e. *sweating*) [6, 7] and a possible increase in *respiratory rate,* caused by sympathetic arousal [7]. Nevertheless, our findings differ sometimes from the results of these two reviews. For instance, our results suggest that mobilizations may have no effect on cardiovascular autonomic activity (very low- to low-certainty evidence) and no effect on *skin temperature*, an outcome which was commonly used to assess skin sympathetic nerve activity.

Contrary to our findings, *Kingston* et al. [7] concluded that mobilizations (oscillatory technique) produced a significant increase in cardiovascular sympathetic activity, as they found an increase in *heart rate* and *blood pressure* in two studies. They also reported, as *Chu* et al. [6] in their meta-analysis, a significant decrease in *skin temperature* compared to a control.

The difference between our results and those reported by *Kingston* et al. [7] might be explained by the inclusion of additional studies in our review, 5 studies dealing with heart rate and 3 studies dealing with blood pressure. The analysis of these new studies is in favor of the absence of an effect.

Inconsistency between our results and those reported by *Chu* et al. [6] might be explained by difference in methodology and data analysis. They performed a meta-analysis mainly based on the difference between mobilizations and an inactive control. Thus the between-group difference is likely to be larger, than when comparing mobilizations to a sham, as in our review. In other words, a proper sham is more likely to induce non-specific effects related to brain-mind responses [22] and thus decreasing the effect (i.e. the difference between the treatment and the sham) than an inactive control, where the study subject knows that nothing happens.

Our results on HVLA manipulation differ from those found in the review of *Amoroso Borges* et al. [8], who reported changes in *parasympathetic and sympathetic nervous system activity* in relation to the treatment area. The reason for this difference is probably that our analysis was more stringent than theirs, since we included only randomized sham-controlled trials and based our results on between-group differences.

### Methodological considerations of our review

A major strength of this review is that we included only randomized sham-controlled trials, which is the preferred design to study the effect of an intervention. Although it is possible that we failed to find all relevant studies, we consider this unlikely as we used a broad search strategy across six databases with no time restriction. Further, we included also French to the usual English language and all reference lists of the included studies were searched for additional studies.

As another strong point, we assessed the certainty of evidence using the Cochrane GRADE approach [24]. This was done by following the classical approach of taking into account factors such as risk of bias, inconsistency, indirectness, imprecision, but also the technical quality of the studies. In addition to the traditional GRADE approach, we assessed the technical quality of the study, as this is an important domain to consider, when assessing evidence in this type of research, as failures in domains such as control of the experimental conditions or in the data acquisition may lead to invalid results.

However, in the absence of a gold standard to assess technical quality we created a topic-specific check-list. The items included in this assessment tool were based on personal experience and knowledge of members of the research team guided by previous research on this topic [26–28], and an external research engineer, specialized in this type of research. We used an arbitrary, but conservative, threshold set at 50% to decide if the studies were technically acceptable or not. It means that we consider reasonable, without being too restrictive, to trust less results from a study which fulfills less than half of the basic technical points recommended in previous literature. The use of a scale with a score to assess quality is sometimes discouraged, as it can be difficult to justify the attributed ‘weights’ to different items. However, we weighted our items (or sub-items) equally. In general, the technical quality of the studies was judged to be acceptable, and thus did not impact our assessment of the certainty of evidence. In three cases, the technical quality of the studies was judged to be a limitation and was, therefore, used to downgrade the certainty of evidence. In only two cases, did this lead to a different (more conservative) conclusion from the classical GRADE approach (without the technical score). Specifically, in these two cases 2/3 studies and 1/3 study obtained a low technical score based on a lack of information in several items, such as whether the experimental conditions were controlled, whether there was a sufficient rest period before baseline recordings, whether an adequate sampling rate was used, or whether a data cleaning process was performed. Thus, in these two cases we believe that the use of the technical check-list for downgrading the certainty of the evidence resulted in more cautious and trustworthy conclusions. Although, in the absence of gold standard, the lack of validation of the technical check-list may be a limitation, we consider its use as a strength of this review that makes it possible to systematically assess important technical points and to suggest technical recommendations for further research.

### Methodological considerations of included studies

Although all our included studies were randomized sham-controlled studies, there were several factors which limit the certainty of evidence. There was, generally, an unclear risk of bias concerning the blinding of the participants, the blinding of the data extraction / cleaning process, and the blinding of the statistician. The certainty of evidence was sometimes downgraded for indirectness, i.e. when studies used outcomes, which are not suitable to appraise autonomic mediated physiology (see below), or because they did not provide some quantitative measures of autonomic activity (e.g. ‘mean’ heart rate instead of heart rate variability).

Generally, included studies did not provide information on the reproducibility or reliability of the measurements (Additional file 3**)**. Thus, we were unable to see if any measurement errors could have affected the results. This topic is also briefly discussed in the recommendation section.

None of the studies testing HVLA manipulation used SC to assess acute changes in skin sympathetic nerve activity, whereas several studies on mobilizations, generally, found a statistically significant effect with this outcome variable. This limits the possibility to make direct comparisons between studies on HVLA techniques and those on joint mobilizations.

In our opinion, it is relevant to consider the possible consequences of different ‘sham’ approaches. Obviously, the choice of a ‘good’ sham procedure in studies dealing with JMT is difficult. In fact, none of the studies, which reported a statistically significant « effect », used a sham which was able to mimic the *mechanical* aspect of the JMT to produce the same level of mechanical stress but outside the joint area. The preferred sham procedure in this field of research is usually described as ‘a manual contact without movement’. This procedure was sufficient to blind the subject in several studies. However, when the sham procedure is relatively ‘inert’ we can reasonably wonder, if the increase in sympathetic activity found with mobilizations (oscillatory technique) is, at least partly, the consequence of a specific mechanism activated by the passive movement of the joint and surrounding tissues. Another possibility is that it might be sufficient to mobilize or pull any other structures in the body outside of a joint area to obtain the same ‘effect’. In other words, unless intervention and sham are basically equal but in one case delivered on the spine / a peripheral joint and the other outside of the spine / the peripheral joint area, it is difficult to determine if the joint component of the technique additionally affects ANS activation or not.

Finally, the vast majority of studies did not assess the potential effect after the immediate post intervention period; thus, we do not know if changes in ANS activation occur after this period and, if so, the direction of such changes.

### Relevance of the findings

Our findings are limited to the acute effect of JMT on markers of ANS activity, while possible treatment effects and interrelations between ANS activity and pain modulation are beyond the scope of this systematic review. As our findings mainly come from fundamental research, their clinical relevance is limited. Nevertheless, these results allow for some clinical considerations.

Traditionally, chiropractors assume that HVLA manipulations have different effects on ANS activity in relation to the anatomical organization of the ANS. Two studies [64, 65], not included in this review, tested this assumption and provided some results to support this theory. However, they suffered from one or several methodological limits (small study sample, lack of randomization, lack of sham or inactive control), for which reasons true effect could not be established.

The evidence at this stage, thus, does not support this use of spinal manipulation, primarily since we found that the spinal HVLA technique may have no acute effect on the studied markers of ANS activity (very low- to low-certainty evidence). Additionally, mobilizations (oscillatory technique), which *did* produce an increase in sympathetic nervous system activity, did so *regardless* of the spinal level that was mobilized.

### Methodological recommendations for future research

In addition to observing the usual pitfalls of bias in experimental and clinical trials, it is also important to consider some purely topic-specific technical aspects of this type of experimentation. Some are described below.

#### Controlled conditions

Because autonomic measurements are sensitive to environmental factors, it is necessary to control for temperature, humidity and to refrain from food, caffeine, alcohol and drug intakes as well as limiting physical activity before the experimentation. It is also very important to use a sufficient rest period before baseline measurements are taken to stabilize ANS activity [27].

#### Choice of outcome

Further, the autonomic system should be measured through several outcomes not to miss changes in an ‘end organ’ activity (see below), including those that are easiest to obtain, such as the mean heart rate and the mean blood pressure. However, this should be done concurrently with more complex computed outcomes such as SC, HRV, arterial blood pressure variability, and baroreflex sensitivity.

In addition, we recommend the use of several « computed » outcome variables to obtain a global assessment of the ANS with SC and systolic blood pressure variability [66] for the sympathetic nervous activity and several parameters of HRV for the vagal modulation of the heart rate. The use of several outcome variables testing ANS activity by measuring the function of different “end organs” is interesting as “there is substantial anatomical and physiological evidence for differential regulation of sympathetic outflow to functionally specific targets” [67]. In other words, multiple outcome variables measuring the activity of several ‘end organs’ may be useful to avoid missing a change in ANS activity and also to better understand the underlying mechanisms.

However, some outcome variables, ‘skin temperature’ and ‘skin blood flow’, may not be well adapted to assess skin sympathetic nerve activity following a JMT. In a review, *Zegarra-Parodi* et al. [28] pointed out that non-sympathetic factors are involved in ‘skin blood flow’ and ‘skin temperature’ regulation and therefore they may not be appropriate to assess skin sympathetic nerve activity.

#### Data acquisition

The acquisition of the complex computed outcome variables must be done with a sufficient sampling rate (using the highest possible if there are no guidelines) and the researchers should be transparent with respect to the management of the raw data.

#### Reliability, reproducibility

Testing for reliability of autonomic mediated physiology outcomes and reproducibility of the findings between studies should be considered in further research, especially those dealing with chronic pain patients. Indeed, failure in these domains would challenge future clinically useful findings.

#### Study design

If the aim is to provide evidence on the difference between specific changes induced by the JMT, to those attributable to the brain-mind responses (e.g. placebo or nocebo), we suggest using randomized sham-controlled trials and to assess with a questionnaire if subjects were well blinded to the intervention or if they had the same expectations for the different interventions [41–44, 47, 50, 55]. Depending on the research question, researchers may consider, in further trials, the use of sham procedures adopting *mechanical* profiles similar to the JMT without the joint component for the reasons stated above. Such a sham procedure may be performed on the scapula or a muscle, as shown by *Budgell* et al. [57, 58]. It is worth noting that a sham procedure, very similar to these recommendations, has recently been validated for successful blinding of the subjects but in another research context [68].

#### Statistical analysis and data reporting

Finally, and very importantly, primary statistical analyses should determine the difference between-groups (e.g. JMT versus Sham) instead of only testing differences from baseline within each group, in agreement with established recommendations for randomized controlled trials [34, 69]. Authors should also report statistical parameters such as means with standard deviations, the mean difference between the intervention and the control as well as the corresponding standard error or confidence interval to facilitate further meta-analysis.

### Perspectives

After having read critically and attempted to interpret the findings in a large number of articles within this field, we are of the opinion that the research on the effects of JMT on markers of ANS activity should be continued but in a more focused manner.

First, in a context of fundamental research, assessment of the effects of JMT on autonomic mediated physiology should be performed over a longer period, as only short-lived effects would limit the relevance of the findings. Considering the relation between pain and autonomic physiology [70, 71], it would be interesting to do so concurrently with the assessment of the effect on experimentally induced pain to evaluate the relationship between pain and autonomic modulations after JMT [16].

Second, this should be performed in a clinical context, assessing the effect of JMT or multimodal manipulative techniques (e.g. JMT combined with muscle release techniques, stretching) on the cardiovascular autonomic activity over the course of several treatment sessions. This is highly interesting, as chronic pain patients may have a disturbed cardiovascular autonomic control [72, 73], and, further, this would make the research more clinically relevant.

## Conclusions

Evidence from 29 randomized sham-controlled trials suggests that one type of joint manipulative technique, mobilizations with oscillatory movements, probably produce an immediate and -at least- short-term bilateral increase in skin sympathetic nerve activity regardless of the area treated (moderate-certainty evidence). This effect may be relevant in a context of fundamental research but, presently, has limited direct clinical relevance. Given the current state of the knowledge from randomized sham-controlled trials, spinal manipulation (HVLA technique) and spinal SNAGs / mobilization with movement may have no acute effect on the studied markers of ANS activity (very low- to low-certainty evidence). Overall, the certainty of evidence was often assessed as very low or low, thus further studies are likely to change these conclusions (but not necessarily the results). Further studies should address the effect of JMT on markers of ANS activity i) in fundamental research settings over a longer duration and concurrently with measures of pain and ii) in people with chronic pain including an assessment of the cardiovascular autonomic control.

## Additional files

Additional file 1: Descriptive check-list. This file contains the main features of each included study. (DOCX 31 kb)

Additional file 2: Risk of bias tool, support for judgment. This file contains the risk of bias tool with support for judgement for each included study. (DOCX 59 kb)

Additional file 3: Technical quality check-list. This file contains the technical quality check-list (scoring system, summary, and details). (DOCX 43 kb)

Additional file 4: Results table. This file contains the results for each included study. (DOCX 40 kb)

## Abbreviations

ANS: Autonomic nervous system
BPM: Beats per minute
HRV: Heart rate variability
HVLA: High-velocity low-amplitude
JMT: Joint manipulative technique(s)
SC: Skin conductance
SNAG: Sustained natural apophyseal glide
