# Supplementary material for: The acute effects of joint manipulative techniques on markers of autonomic nervous system activity: a systematic review and meta-analysis of randomized sham-controlled trials
Source: Chiropr Man Therap. 2019 Mar 12;27:17. doi: 10.1186/s12998-019-0235-1 (PMC6413458; doi:10.1186/s12998-019-0235-1)
Supplement: Supplementary file 1 — Descriptive check-list. This file contains the main features of each included study. (DOCX 31 kb) [file 12998_2019_235_MOESM1_ESM.docx]

**Additional file 1**

**Descriptive check-list**

| **Descriptive check-list**  **Mobilizations (oscillatory technique)** | | | | | | | | |
| --- | --- | --- | --- | --- | --- | --- | --- | --- |
| **Author**  **year**  **country** | **Design** | **Source of participants** | **Symptoms** | **Age of participants**  Range & means | **no of subjects**  **1/ recruited**  **2/ analyzed**  **(Female / Male)** | **Interventions** | **Treatment area**  **- “lesion” (**restriction of mobility or pain)  **- non “lesion”** | **Outcome variables**  Main outcome variables related to autonomic mediated physiology |
| Petersen  1993  Australia  [35] | Crossover RCT | ? | no | Range 18-35  Mean:? | 1/ N: 16  (0/16)  2/ N: 16? | Joint manipulative technique: Grade III postero-anterior mobilization (central pressure)  Sham/Placebo: same contact without movement.  *Both interventions applied for 3×1 minute with a rest period between applications of 1 minute*  Control: no contact | C_5_ “non-lesion” | Skin conductance (right hand)  Skin temperature (right hand) |
| Vicenzino  1994  Australia  [36] | Crossover RCT | Staff and students from university | no | Range:?  Mean: 26 | 1/ N: 34  (14/20)  2/ N: 34? | Joint manipulative technique 1: Grade III left lateral glide mobilization + ULTT1  Joint manipulative technique 2: Grade III left lateral glide mobilization + ULTT2  Sham/Placebo: same contact without mobilization + no nerve tension test  *Both interventions applied for 3×30 sec with a rest period between applications of 1 minute*  Control: no contact | C_5_ / C_6_ “non-lesion” | Skin conductance (both hands)  Skin temperature (both hands) |
| Slater  1994  Australia  [37] | Crossover RCT | ? | no | Range:?  Mean: 24 | 1/N: 22  (8/14)  2/ N: 22? | Joint manipulative technique: Grade IV postero-anterior mobilization in “sympathetic slump” position  Sham/Placebo light touch in long sitting position  *Both interventions applied for 3×20 sec with a rest period between applications of 1 minute*  Control: no contact | T_6_ right costo-vertebral joint “non-lesion” | Skin conductance (both hands)  Skin temperature (both hands) |
| Simon  1997  Australia  [38] | Crossover RCT | ? | no | Range: 16 to 25  Mean: 19,5 ± 2 | 1/ 19  (13/6)  2/ N: 19? | Joint manipulative technique: Grade III antero-posterior mobilization  Sham/Placebo: same procedure without mobilization  *Both interventions applied for 3×30 sec with a rest period between applications of 1 minute*  Control: no contact | Right glenohumeral joint “non-lesion” | Skin conductance (both hands)  Skin temperature (both hands) |
| RCT: randomized-controlled trial; PPT: pressure pain threshold | | | | | | | | |

| **Descriptive check-list**  **Mobilizations (oscillatory technique)** | | | | | | | | |
| --- | --- | --- | --- | --- | --- | --- | --- | --- |
| McGuiness  1997  Australia  [39] | Crossover RCT | ? | no | Range: 18-29  Mean:? | 1/ N: 23  (11/12)  2/ N: 23? | Joint manipulative technique : Grade III postero-anterior mobilization  Sham/Placebo: contact without movement  *Both interventions applied for 3×1 minute with a rest period between applications of 1 minute*  Control: no contact | C_5_ “non-lesion” | Heart rate  Respiratory rate  Blood pressure |
| Vicenzino  1998  Australia  [40] | Crossover RCT | ? | no | Range 17-35  Mean: 21 ± 4,7 | 1/ N: 24  (13/11)  2/ N: 24? | Joint manipulative technique: Grade III left lateral glide mobilization + ULTT 2  Sham/Placebo:?  *Both interventions applied for 3×30 sec with a rest period between applications of 1 minute*  Control:? | C_5_ “non-lesion” | Heart rate  Respiratory rate  Blood pressure (left arm) |
| Vicenzino  1998  Australia  [41] | Crossover RCT | ? | Unilateral chronic lateral epicondylalgia | Range: 27-70  Mean: 49 ± 10 | 1/ N: 24  (13/11)  2/ N: 24 | Joint manipulative technique: Grade III oscillatory lateral glide mobilization  Sham/Placebo: contact without movement  *Both interventions applied for 3×30 sec with a rest period between applications of 1 minute*  Control: no contact | C_5_ / C_6_ “non-lesion” | Skin conductance (affected limb)  Skin temperature (affected limb)  Skin blood flux (affected limb) |
| Sterling  2001  Australia  [42] | Crossover RCT | ? | History of mid or lower cervical spine pain ≥ 3 months | Range:?  Mean: 36 | 1/ N: 30  2/ N: 30  (16/14) | Joint manipulative technique: Grade III postero-anterior mobilization  Sham/Placebo: contact without movement  *Both interventions applied for 3×1 minute with a rest period between applications of 1 minute*  Control: no contact | C_5_ / C_6_ symptomatic side “lesion” | Skin conductance (both hands)  Skin temperature (both hands) |
| ULTT: upper limb tension test | | | | | | | | |

| **Descriptive check-list**  **Mobilizations (oscillatory technique)** | | | | | | | | |
| --- | --- | --- | --- | --- | --- | --- | --- | --- |
| Perry  2008  UK  [43] | Independent group RCT | ? | no | Range :18-25  Mean: 21.5  Mobilization: 21.4  Sham: 21.5  Control: 21.7 | 1/ N: 45  (0/45)  2/ N: 45  (15 per group) | Joint manipulative technique:  Grade III oscillatory mobilization at 2 Hz  Sham/Placebo: light pressure without oscillatory movement  *Both interventions applied for 3× 1 minute with a rest period between applications of 1 minute*  Control: no contact | Left L_4_ / L_5_ facet joint “non-lesion” | Skin conductance (both feet) |
| Jowsey  2010  UK  [44] | Independent group RCT | ? | no | Range :18-35  Mean: 22.7 ± 5  Mobilization  Mean: 23 ± 6  Sham/placebo:  Mean: 22 ± 4.3 | 1/ N: 36  (23/13)  2/ N: 36 | Joint manipulative technique: Grade III rotatory postero-anterior mobilization at 0.5 Hz  Sham/placebo: pressure without oscillation  *Both intervention applied for 3×1 minute with a rest period between applications of 1 minute* | T4 « non-lesion » | Skin conductance (Both hands) |
| La Touche  2013  Spain  [45] | Independent groups RCT | Patients from 2 private dental clinics and 3 universities | Chronic craniofacial pain from myofascial origin (pain and dysfunction located at the cervical and masticatory muscles) | Mobilization  Range:?  Mean: 33.2 ± 9,5  Sham group  Range:?  Mean: 34.5 ± 8 | 1/ N: 32  (21/11)  2/ N: 32 | 3 treatment sessions over 2 weeks  Joint manipulative technique: Antero-posterior upper cervical mobilization  at 0.5 Hz  Sham /placebo: Same contact without mobilization  *Both intervention applied for 3×2 minutes with a rest period between applications of 30 sec.* | C0 - C3 “lesion” | Skin conductance (left hand)  Skin temperature (left hand)  Heart rate  Breathing rate |
| Yung  2014  USA  [46] | Independent groups RCT | ? | no | Mobilization:  range:  mean: 24.6 ± 2.2  Sham group  range:  mean: 24.8 ± 1.6 | 1/ N: 39  (54 screened)  2/ N: 39  (25/14) | Joint manipulative technique:  Antero-posterior mobilization *at* 1.5 Hz  *5 sets of 10 sec of AP pressure with 10 sec rest between sets*  Sham /placebo: light touch | C6 “non-lesion” | Heart rate  Blood pressure |

| **Descriptive check-list**  **Mobilizations (oscillatory technique)** | | | | | | | | |
| --- | --- | --- | --- | --- | --- | --- | --- | --- |
| Piekarz  2016  UK  [47] | Independent groups RCT | Student population from university | no | Range: 18-25  mean: 21.5  Mobilization:  Mean: 22 ± 2 and 21 ± 2  Sham/placebo:  Mean: 22 ± 2  Control:  Mean: 22 ± 2 | 1/ N: 60  (67 assessed)  2/ N: 60  (0/60) | Joint manipulative technique :  - 3Hz Maitland mobilization (94-109 N)  - 2Hz Maitland mobilization (94-103 N)  Sham/Placebo: same contact + pressure (mean: 101 N) without oscillatory movement  *Interventions applied for 3×1 min with a rest period between applications of 1 minute*  Control: no manual contact | L4 “non-lesion” | Skin conductance (both feet) |
| Zegarra-Parodi  2016  USA  [48] | Crossover RCT | Participant in a university setting | no | Mean: 25 ± 5,5 | 1/?  2/32  (21/11) | Joint manipulative technique:  -High pressure mobilization (80% of the PPT) 0.5 Hz  -Low pressure mobilization (40% of the PPT) 0.5 Hz  Sham/Placebo mobilization (5 % of the PPT) 0.5 Hz  I*ntervention applied for 3× 1 minute with a rest period between applications of 1 minute.*  Control: no contact | T1 (side of the dominant Hand) “non-lesion” | Skin Blood Flux (both hands)  Skin temperature (non-dominant hand)  Blood pressure  Heart Rate |
| Yung  2017  [49] | Independent groups RCT | Staff, faculty, students from 2 universities | no | Range:  Mean: 23.8 ± 3  Mobilization:  Mean: 24 ± 4  Sham/placebo:  Mean: 24 ± 2 | 1/ N: 44  (46 screened)  2/ N: 44  (18/26) | Joint manipulative technique:  Postero-anterior mobilization at 1.5 Hz  *5 sets of 10 sec of PA pressures with a rest period between applications of 10 sec*  Sham /placebo: light touch | C6 « non-lesion » | Heart rate  Blood pressure |
| Araujo  2017  Brazil  [50] | Independent groups RCT | ? | no | Range:  Mean: 22. ± 4  Mobilization:  Mean: 22. ± 4  Mobilization + slump:  Mean: 22.3 ± 2.5  Sham/placebo:  Mean: 23 ± 5 | 1/ N: 60  (86 screened)  2/ N: 57  (39/18) | Joint manipulative technique 1:  Grade III posterior-to-anterior rotatory passive accessory intervertebral mobilization at 2 Hz  Joint manipulative technique 2:  unilateral grade III posterior-to-anterior rotatory passive accessory intervertebral mobilization at 2 Hz + slump  Sham/Placebo: only manual contact without mobilization  *Interventions applied for 3× 1 minute with a rest period between applications of 1 minute* | T4 “non-lesion” | Heart rate variability  Heart rate |
| RCT: randomized-controlled trial; PPT: pressure pain threshold | | | | | | | | |
| **Mobilizations (atypical technique))** | | | | | | | | |
| Henderson  2010  USA  [51] | Independent group RCT | University campus | no | Invited range: 21-60  Included mean: 30.9 | 1/ N: 23  2/ N: 14 (7 per group)  (9/5) | Joint manipulative technique: Rib raising mobilization  Sham/Placebo: light touch (no lifting was done)  *Both interventions applied for 3×5 cycles on each side* | T1-T4, T5-T8, T9-T12 “non-lesion” | Salivary amylase  Salivary flow rate |

| **Descriptive check-list**  **Mobilizations (SNAGs / mobilization with movement)** | | | | | | | | |
| --- | --- | --- | --- | --- | --- | --- | --- | --- |
| Paungmali  2003  Australia  [52] | Crossover RCT | From both general population and local health care practitioners | Unilateral lateral epicondylalgia | Range:?  Mean: 48,5 ± 7 | 1/ N: 24  2/ N: 24  (7/17) | Joint manipulative technique: Mobilization with movement  *10 repetitions sustained 6 secs with 15 secs at rest between*  Sham/Placebo: contact without glide  Control: no manual force applied | Elbow: “lesion” | Skin conductance (both hands)  Skin temperature (both limbs)  Cutaneous blood flow (affected limb)  Blood pressure (unaffected limb)  Heart rate |
| Moulson  2006  UK  [53] | Crossover RCT | ? | no | Range: 18 - 37  Mean: 23 ± 5 | 1/ N: 16  2/ N: 16  (11/5) | Joint manipulative technique:  Mulligan’s sustained natural apophyseal glides with active head rotation (3 times)  Sham/Placebo: same procedure without glide  Control: no contact | C5/C6 “non-lesion” | Skin conductance (both hands)  Skin temperature *(*both hands) |
| Moutzouri  2012  UK  [54] | Independent groups RCT | From university | no | Treat:  Range 18 - 46  Mean: 25 ± 8  Sham/placebo:  Range 19 - 43  Mean: 27 ± 8  Control:  Range 18 - 45  Mean: 27 ± 10 | 1/ N: 45  (55 screened)  2/ N: 45  (33/12) | Joint manipulative technique: Mulligan’s sustained natural apophyseal glides (centrally applied) while participant performed 6 repetitions of full active lumbar flexion in sitting.  Sham/Placebo: same procedure without SNAG  *Both with 3 sets of 6 repetitions with 1 minute rest between*  Control: no contact | L4 “non-lesion” | Skin Conductance (both feet) |
| Tsirakis  2015  UK  [55] | Independent groups RCT | Student from university | no | Range: 18 - 35  Mean: 23.6 ± 4,5  Treat:  Mean: 24 ± 5  Sham/placebo:  Mean: 23,5 ± 4,5  Control:  Mean: 23,5 ± 4,5 | 1/ N: 45  (52 assessed)  2/ N: 45  (0/45) | Joint manipulative technique: Mulligan’s sustained medial glides with passive right leg flexion movements (repeated 3 times)  Sham/Placebo: lumbar contact without glide + leg slightly lifted by the belt without any movement)  Control: no manual contact | L4 unilateral lamina “non-lesion” | Skin conductance (both feet) |
| Bowler  2017  UK  [56] | Crossover RCT | People from university | no | Range: 18 - 48  Mean: 30 | 1 /N: 30  2/ N: 30  (19/11) | Joint manipulative technique:  Ipsilateral or contralateral Mulligan’s sustained natural apophyseal glides with active head rotation (10 times)  Sham/placebo: contact without glide  Control: no manual contact | C5 right (ipsilateral technique) or left (contralateral technique) articular pillar | Skin conductance (right hand)  Skin temperature *(*right hand) |
| SNAG: sustained natural apophyseal glide | | | | | | | | |

| **Descriptive check-list**  **HVLA manipulation** | | | | | | | | |
| --- | --- | --- | --- | --- | --- | --- | --- | --- |
| Budgell  2001  [57] | Crossover RCT | ? | no  (at most a trivial level of neck discomfort) | Range: 21 to 40  Mean: 28,5 ± 6 | 1/ N: 25  (5/20)  2/ N: 24 | Joint manipulative technique :  HVLA manipulation  Sham/placebo :  Same procedure but thrust on the skin | C1 /C2 “non-lesion” | Heart rate variability  Heart rate |
| Budgell  2006  [58] | Crossover RCT | First-year students in a health sciences program | no  (at most a trivial level of cervicothoracic discomfort) | Range: 18 to 45  mean: 29 ± 7 | 1/ N: 31  2/ N: 28  (5/23) | Joint manipulative technique:  HVLA manipulation  Sham/placebo: Thrust on scapula | T1 – T4 with a restriction of movements “lesion”  Scapula for the Sham | Heart rate variability  Heart rate |
| Roy  2009  Canada  [59] | Independent groups  RCT | Patients from different chiropractic clinic | Acute low back pain | Range:?  Mean Treat: 36 ± 12  Mean Sham: 45 ± 10 | 1/ N: 20  (12/8)  2/ N: 20 | Joint manipulative technique :  HVLA manipulation  Sham/placebo :  5 sec pressure without thrust | L5 “lesion” | Heart rate variability |
| Sillevis  2010  USA  [60] | Independent groups  RCT | Patients from 5 outpatient physical therapy clinics | Chronic cervical pain | Invited: 18-65  Mean Treat: 43  Mean Sham: 47 | 1/ N: 101  (135 assessed)  2/ N: 100  (77/23) | Joint manipulative technique :  HVLA manipulation  Sham/placebo :  same contact + pressure without thrust | T3 / T4 “non-lesion” | Pupil diameter |
| Puhl  2012  Canada  [61] | Independent groups  RCT | Staff, students from CMCC as well as their friends and relatives | no | Invited: 20 to 45  Mean Treat: 26  Mean Sham: 26 | 1/ N: 56  2/ N :36  (17/19) | Joint manipulative technique:  HVLA manipulation  Sham/placebo: contact without thrust | T1-T6 hypomobile segment « lesion » | Plasma norepinephrine  Plasma epinephrine |
| Ward  2013  USA  [62] | Independent groups  RCT | Chiropractic students | no | Range:?  Mean Treat: 27 ± 3,  Mean Sham: 26,5 ± 6,5  Mean Control :25,5 ± 1,5 | 1/N: 36  (48 assessed)  2/N: 36  (18/18) | Joint manipulative technique:  HVLA manipulation  Sham/Placebo: activator with no force  Control: no manual contact | T1- T4 “non-lesion” | Heart rate  Bilateral Blood pressure |
| Sampath  2017  New Zealand  [63] | Independent groups  RCT | General population | no | Invited: 18 to 45  Mean Treat: 28  Mean Sham: 27 | 1/ N: 24  (37 assessed)  2/ N: 24  (0/24) | Joint manipulative technique:  HVLA manipulation  Sham: contact without thrust | T5 | Heart rate variability  Near infrared spectroscopy: oxy-hemoglobin |
| HVLA: high velocity low amplitude | | | | | | | | |
