# Supplementary material for: The acute effects of joint manipulative techniques on markers of autonomic nervous system activity: a systematic review and meta-analysis of randomized sham-controlled trials
Source: Chiropr Man Therap. 2019 Mar 12;27:17. doi: 10.1186/s12998-019-0235-1 (PMC6413458; doi:10.1186/s12998-019-0235-1)
Supplement: Supplementary file 2 — Risk of bias tool, support for judgment. This file contains the risk of bias tool with support for judgement for each included study. (DOCX 59 kb) [file 12998_2019_235_MOESM2_ESM.docx]

**Additional file 2**

**Risk of bias tool, support for judgement**

| **Risk of Bias tool: Petersen 1993** | | |
| --- | --- | --- |
| **Bias** | **Authors' judgement** | **Support for judgement** |
| Random sequence generation (selection bias) | - Low risk of bias | “researcher B, who drew lots to determine the order for each subject” |
| Allocation concealment (selection bias) | - Unclear risk of bias | Insufficient information |
| Blinding of participants and personnel (performance bias) | - Unclear risk of bias | “double blind design”  Comment: not tested with a questionnaire |
| Blinding of outcome assessment (detection bias) | - Low risk of bias | “researcher A was blind to the procedure” |
| Incomplete outcome data (attrition bias) | - Unclear risk of bias | Insufficient information |
| Selective reporting (reporting bias) | - Unclear risk of bias | Insufficient information |
| Other bias:  Blinding of subjects to apparatus display | - Low risk of bias | “A screen was placed between the subject and the recording equipmen such that the subject and the researcher B were blind to the recordings” |
| Other bias:  Blinding of therapist to apparatus display | - Low risk of bias | “A screen was placed between the subject and the recording equipmen such that the subject and the researcher B were blind to the recordings” |
| Other bias:  Blinding of the data extraction / cleaning process | Irrelevant | Irrelevant  “Researcher A…noted SC and St value at 15 sec intervals” |
| Other bias:  Blinding of the statistician | - Unclear risk of bias | Insufficient information |

| **Risk of Bias tool: Vicenzino 1994** | | |
| --- | --- | --- |
| **Bias** | **Authors' judgement** | **Support for judgement** |
| Random sequence generation (selection bias) | - Unclear risk of bias | “Patients were randomly assigned to an order of treatment conditions” |
| Allocation concealment (selection bias) | - Unclear risk of bias | Insufficient information |
| Blinding of participants and personnel (performance bias) | - Unclear risk of bias | “double blind, placebo controlled”  Comment : not tested with a questionnaire |
| Blinding of outcome assessment (detection bias) | - Low risk of bias | “The barrier between the treatment cubicle and the recording room facilitated the experimental blinding of researcher A and therapist” |
| Incomplete outcome data (attrition bias) | - Unclear risk of bias | Insufficient information |
| Selective reporting (reporting bias) | - Unclear risk of bias | Insufficient information |
| Other bias:  Blinding of subjects to apparatus display | - Low risk of bias | “Visual and auditory outpout of these devices were disabled so that both therapist and subject were blinded to the effect of the treatment conditions” |
| Other bias:  Blinding of therapist to apparatus display | - Low risk of bias | “Visual and auditory outpout of these devices were disabled so that both therapist and subject were blinded to the effect of the treatment conditions” |
| Other bias:  Blinding of the data extraction / cleaning process | - Unclear risk of bias | “Researcher A monitored the in-coming data”  Insufficient information |
| Other bias:  Blinding of the statistician | - Unclear risk of bias | Insufficient information |

| **Risk of Bias tool: Slater 1994** | | |
| --- | --- | --- |
| **Bias** | **Authors' judgement** | **Support for judgement** |
| Random sequence generation (selection bias) | - Unclear risk of bias | “Patients were randomly assigned to an order of treatment conditions” |
| Allocation concealment (selection bias) | - Unclear risk of bias | Insufficient information |
| Blinding of participants and personnel (performance bias) | - Unclear risk of bias | “double blind nature of the experiment”  Comment : not tested with a questionnaire |
| Blinding of outcome assessment (detection bias) | - Low risk of bias | “the researcher responsible for the data acquisition was located in an adjoining laboratory” |
| Incomplete outcome data (attrition bias) | - Unclear risk of bias | Insufficient information |
| Selective reporting (reporting bias) | - Unclear risk of bias | Insufficient information |
| Other bias :  Blinding of subjects to apparatus display | - Low risk of bias | “Subject and the therapist applying the treatment could not see any of the recorded data” |
| Other bias :  Blinding of Therapist to apparatus display | - Low risk of bias | “Subject and the therapist applying the treatment could not see any of the recorded data” |
| Other bias:  Blinding of the data extraction / cleaning process | - Unclear risk of bias | Insufficient information |
| Other bias  Blinding of the statistician | - Unclear risk of bias | Insufficient information |

| **Risk of Bias tool: Simon 1997** | | |
| --- | --- | --- |
| **Bias** | **Authors' judgement** | **Support for judgement** |
| Random sequence generation (selection bias) | - Unclear risk of bias | “the study used a randomized repeated measures design” |
| Allocation concealment (selection bias) | - Unclear risk of bias | Insufficient information |
| Blinding of participants and personnel (performance bias) | - Unclear risk of bias | “in order to maintain double blind conditions”  Comment : not tested with a questionnaire |
| Blinding of outcome assessment (detection bias) | - Low risk of bias | “the computer and the investigator responsible for data collection were located in an adjacent laboratory” |
| Incomplete outcome data (attrition bias) | - Unclear risk of bias | Insufficient information |
| Selective reporting (reporting bias) | - Unclear risk of bias | Insufficient information |
| Other bias :  Blinding of subjects to apparatus display | - Low risk of bias | “the computer and the investigator responsible for data collection were located in an adjacent laboratory” |
| Other bias :  Blinding of Therapist to apparatus display | - Low risk of bias | “the computer and the investigator responsible for data collection were located in an adjacent laboratory” |
| Other bias :  Blinding of the data extraction / cleaning process | - Unclear risk of bias | Insufficient information |
| Other bias  Blinding of the statistician | - Unclear risk of bias | Insufficient information |

| **Risk of Bias tool: McGuiness 1997** | | |
| --- | --- | --- |
| **Bias** | **Authors' judgement** | **Support for judgement** |
| Random sequence generation (selection bias) | - Unclear risk of bias | “randomized…design”  “and randomized the order of testing” |
| Allocation concealment (selection bias) | - Unclear risk of bias | Insufficient information |
| Blinding of participants and personnel (performance bias) | - Unclear risk of bias | “Subjects blinding was ensured by recruiting volunteers with no experience of manual therapies”  Comment : not tested with a questionnaire |
| Blinding of outcome assessment (detection bias) | - Low risk of bias | “the third investigator was blind to the experimental condition”  Comment : No information about the second investigator |
| Incomplete outcome data (attrition bias) | - Unclear risk of bias | Insufficient information |
| Selective reporting (reporting bias) | - Unclear risk of bias | Insufficient information |
| Other bias:  Blinding of subjects to apparatus display | - Low risk of bias | “The door between the rooms was closed during testing”  Comment : probably blinded |
| Other bias:  Blinding of herapist to apparatus display | - Low risk of bias | “manipulative therapist was blind to the data recorded” |
| Other bias:  Blinding of the data extraction / cleaning process | - Unclear risk of bias | Insufficient information |
| Other bias:  Blinding of the statistician | - Unclear risk of bias | Insufficient information |

| **Risk of Bias tool: Vicenzino 1998 (cardiovascular)** | | |
| --- | --- | --- |
| **Bias** | **Authors' judgement** | **Support for judgement** |
| Random sequence generation (selection bias) | - Unclear risk of bias | “in a randomized order” |
| Allocation concealment (selection bias) | - Unclear risk of bias | Insufficient information |
| Blinding of participants and personnel (performance bias) | - Low risk of bias | “A post experiment questionnaire was administred to determine the effect of these procedures in blinding the subject to the objective of the study” – “seven subjects have identified the session in which they had received the treatment technique, only one subject had identified that the true objective of the study was”  Comment: benefit of the doubt |
| Blinding of outcome assessment (detection bias) | - Low risk of bias | Comment : Automated and computerized measures for respiratory rate and heart rate. Insuficient information concerning blood pressure measures. |
| Incomplete outcome data (attrition bias) | - Unclear risk of bias | Insufficient information |
| Selective reporting (reporting bias) | - Unclear risk of bias | Insufficient information |
| Other bias :  Blinding of subjects to apparatus display | - Low risk of bias | “subjects were unable to see the displays which were used to record blood pressure, heart rate and respiratory rate” |
| Other bias :  Blinding of therapist to apparatus display | - Low risk of bias | “The therapist was also blind to these measures” |
| Other bias :  Blinding of the data extraction / cleaning process | - Unclear risk of bias | Insufficient information |
| Other bias  Blinding of the statistician | - Unclear risk of bias | Insufficient information |

| **Risk of Bias tool: Vicenzino 1998** | | |
| --- | --- | --- |
| **Bias** | **Authors' judgement** | **Support for judgement** |
| Random sequence generation (selection bias) | - Unclear risk of bias | “In a randomized order” |
| Allocation concealment (selection bias) | - Unclear risk of bias | Insufficient information |
| Blinding of participants and personnel (performance bias) | - Low risk of bias | “the double-blind status of this experiment was ensured in the same manner as in our previous studies…assessed by a postexperiment questionnaire”- “Only one of 24 subjects correctly identified the treatment session” |
| Blinding of outcome assessment (detection bias) | - Low risk of bias | “the double-blind status of this experiment was ensured in the same manner as in our previous studies”  Comment : probably done |
| Incomplete outcome data (attrition bias) | - Unclear risk of bias | Insufficient information |
| Selective reporting (reporting bias) | - Unclear risk of bias | Insufficient information |
| Other bias :  Blinding of subjects to apparatus display | - Low risk of bias | “the double-blind status of this experiment was ensured in the same manner as in our previous studies”  Comment : probably done |
| Other bias :  Blinding of therapist to apparatus display | - Low risk of bias | “the double-blind status of this experiment was ensured in the same manner as in our previous studies”  Comment : probably done |
| Other bias :  Blinding of the data extraction / cleaning process | - Unclear risk of bias | Insufficient information |
| Other bias  Blinding of the statistician | - Unclear risk of bias | Insufficient information |

| **Risk of Bias tool: Sterling 2001** | | |
| --- | --- | --- |
| **Bias** | **Authors' judgement** | **Support for judgement** |
| Random sequence generation (selection bias) | - Low risk of bias | “in a randomized order, established by drawing lots” |
| Allocation concealment (selection bias) | - Unclear risk of bias | Insufficient information |
| Blinding of participants and personnel (performance bias) | - Low risk of bias | “double blind, placebo controlled” – “the effectivemeness of this procedure was evaluated by a post-experiment questionnaire” |
| Blinding of outcome assessment (detection bias) | - Low risk of bias | “Researcher A record all pre and post-experimental measures and was blind to the experimental condition applied, being located in a separate room”  Comment : automated measures for skin conductance and skin temperature” |
| Incomplete outcome data (attrition bias) | - Unclear risk of bias | Insufficient information |
| Selective reporting (reporting bias) | - Unclear risk of bias | Insufficient information |
| Other bias :  Blinding of subjects to apparatus display | - Low risk of bias | Comment: subject in prone position |
| Other bias :  Blinding of therapist to apparatus display | - Low risk of bias | “researcher B was blind to data collected” |
| Other bias :  Blinding of the data extraction / cleaning process | - Unclear risk of bias | Insufficient information |
| Other bias  Blinding of the statistician | - Unclear risk of bias | Insufficient information |

| **Risk of Bias tool: Perry 2008** | | |
| --- | --- | --- |
| **Bias** | **Authors' judgement** | **Support for judgement** |
| Random sequence generation (selection bias) | - Low risk of bias | “subject were randomly assigned to one of the three subject groups using the third party, concealed randomization method” |
| Allocation concealment (selection bias) | - Unclear risk of bias | “subject were randomly assigned to one of the three subject groups using the third party, concealed randomization method” |
| Blinding of participants and personnel (performance bias) | - Low risk of bias | “success of blinding…was invistagated with a previously validated questionnaire” - “a comparison of the treatment and placebo groups revealed that there was no significant difference. Therefore, the subject were unable to distinguish between the genuine treatment and the placebo condition” |
| Blinding of outcome assessment (detection bias) | - Low risk of bias | “the equipment was installed in a screened area adjacent to the treatment plinth, therefore blinding the data collector to the intervention being undertaken and to the hypothesis being tested”. |
| Incomplete outcome data (attrition bias) | - Low risk of bias | “recruited a convenience sample of 45 healthy subjects”  “all 45 subjects completed the study” |
| Selective reporting (reporting bias) | - Unclear risk of bias | Insufficient information |
| Other bias :  Blinding of subjects to apparatus display | - Low risk of bias | “neither the subjects nor the researcher received anyfeedback from the data collector regarding SNS activity” |
| Other bias :  Blinding of therapist to apparatus display | - Low risk of bias | “neither the subjects nor the researcher received anyfeedback from the data collector regarding SNS activity” |
| Other bias :  Blinding of the data extraction / cleaning process | - Unclear risk of bias | Insufficient information  “blinding the data collector to the intervention being undertaken and to the hypothesis being tested, thereby eliminating any biais due to expectations, predictions or preferred outcome”. |
| Other bias  Blinding of the statistician | - Unclear risk of bias | Insufficient information |

| **Risk of Bias tool: Jowsey 2010** | | |
| --- | --- | --- |
| **Bias** | **Authors' judgement** | **Support for judgement** |
| Random sequence generation (selection bias) | - Unclear risk of bias | “subjects were randomly allocated to either…” |
| Allocation concealment (selection bias) | - Unclear risk of bias | Insufficient information |
| Blinding of participants and personnel (performance bias) | - Low risk of bias | “the questionnaire showed that the credibility of the placebo intervention was at least as good as the credibility of the treatment intervention”. |
| Blinding of outcome assessment (detection bias) | - Low risk of bias | “an independent assessor gathered the data”  Comment : well done in a previous study |
| Incomplete outcome data (attrition bias) | - Low risk of bias | “a convenience sample of 36 healthy subjects was used”  Comment : results table shows data from 36 subjects |
| Selective reporting (reporting bias) | - Unclear risk of bias | Insufficient information |
| Other bias :  Blinding of subjects to apparatus display | - Low risk of bias | Comment: probably (picture) |
| Other bias :  Blinding of therapist to apparatus display | - Unclear risk of bias | Insufficient information |
| Other bias :  Blinding of the data extraction / cleaning process | - Unclear risk of bias | Insufficient information |
| Other bias  Blinding of the statistician | - Unclear risk of bias | Insufficient information |

| **Risk of Bias tool: La Touche 2013** | | |
| --- | --- | --- |
| **Bias** | **Authors' judgement** | **Support for judgement** |
| Random sequence generation (selection bias) | - Low risk of bias | “Randomization was performed by a computer generated random-sequence table” |
| Allocation concealment (selection bias) | - Unclear risk of bias | Insufficient information |
| Blinding of participants and personnel (performance bias) | - Unclear risk of bias | “patients were blind to which intervention they received”  Comment : not tested with a questionnaire |
| Blinding of outcome assessment (detection bias) | - Low risk of bias | “an independent assessor, blind to intervention assignement made the measurements and registered the data” |
| Incomplete outcome data (attrition bias) | - Low risk of bias | “No patients dropped out during the study” |
| Selective reporting (reporting bias) | - Unclear risk of bias | Insufficient information |
| Other bias :  Blinding of subjects to apparatus display | - Unclear risk of bias | Insufficient information |
| Other bias :  Blinding of therapist to apparatus display | Irrelevant | Not relevant |
| Other bias :  Blinding of the data extraction / cleaning process | - Unclear risk of bias | “independent assessor blind to intervention assignement made the measurements and registered the data”  Comment: Insufficient information |
| Other bias  Blinding of the statistician | - Unclear risk of bias | Insufficient information |

| **Risk of Bias tool: Yung 2014** | | |
| --- | --- | --- |
| **Bias** | **Authors' judgement** | **Support for judgement** |
| Random sequence generation (selection bias) | - Low risk of bias | “two assistants generated a random allocation sequence from a, online randomizer…and randomly assigned to each participant a sequential number and a random allocation number...” |
| Allocation concealment (selection bias) | - Low risk of bias | “…that was concealed from the therapist and the participants throughout the study” |
| Blinding of participants and personnel (performance bias) | - Unclear risk of bias | Comment : credibility of the placebo procedure was not tested |
| Blinding of outcome assessment (detection bias) | - Unclear risk of bias | No information |
| Incomplete outcome data (attrition bias) | - Low risk of bias | Comment : 39 subjects randomized, 39 subjects analyzed. |
| Selective reporting (reporting bias) | - Unclear risk of bias | Insufficient information |
| Other bias :  Blinding of subjects to apparatus display | - Low risk of bias | “throughout the entire data-collection process both the participants and therapists providing the intervention were blinded to the cardiovascular outcomes assessment” |
| Other bias :  Blinding of therapist to apparatus display | - Low risk of bias | “throughout the entire data-collection process both the participants and therapists providing the intervention were blinded to the cardiovascular outcomes assessment” |
| Other bias :  Blinding of the data extraction / cleaning process | Irrelevant | Irrelevant |
| Other bias  Blinding of the statistician | - Unclear risk of bias | Insufficient information |

| **Risk of Bias tool: Piekarz 2016** | | |
| --- | --- | --- |
| **Bias** | **Authors' judgement** | **Support for judgement** |
| Random sequence generation (selection bias) | - Low risk of bias | “the online number generator randomized the sequence of 60 numbers, which were divided at random in the four treatment groups…” |
| Allocation concealment (selection bias) | - Low risk of bias | “…with results concealed in an opaque envelope. The envelope was only opened on the day of data collection” |
| Blinding of participants and personnel (performance bias) | - Low risk of bias | “Participants remained blind to their intervention group allocation, and success of this was determined with previously validated post trial questionnaire” – “the participant in the placebo, 2hz, 3hz groups were unable to determine differences between interventions” |
| Blinding of outcome assessment (detection bias) | - Low risk of bias | “using a screen to separate the data collector from the treating therapist and participant, so the data collector remained blind” |
| Incomplete outcome data (attrition bias) | - Low risk of bias | “in all 60 participants the biopac system software was used to process the data and perform statistical analysis”  Comment : 67 screened, 60 included |
| Selective reporting (reporting bias) | - Unclear risk of bias | Insufficient information |
| Other bias :  Blinding of subjects to apparatus display | - Low risk of bias | “using a screen to separate the data collector (and SNS display) from the treating therapist and participant” |
| Other bias :  Blinding of therapist to apparatus display | - Low risk of bias | “using a screen to separate the data collector (and SNS display) from the treating therapist and participant” |
| Other bias  Blinding of the data extraction / cleaning process | - Low risk of bias | “the blind data assessor selected from the graphical display the 1-min period within…” |
| Blinding of the statistician | - Unclear risk of bias | Insufficient information |

| **Risk of Bias tool: Zegarra-Parodi 2016** | | |
| --- | --- | --- |
| **Bias** | **Authors' judgement** | **Support for judgement** |
| Random sequence generation (selection bias) | - Low risk of bias | “participants were randomly assigned to 1 of 4 sequences of intervntions following a Williams design, which is a Latin square design balanced with respect to first order carry-over effect” |
| Allocation concealment (selection bias) | - Unclear risk of bias | Insufficient information |
| Blinding of participants and personnel (performance bias) | - Unclear risk of bias | Comment : not tested with a questionnaire |
| Blinding of outcome assessment (detection bias) | - Low risk of bias | Comments: Use of automated measures |
| Incomplete outcome data (attrition bias) | - Low risk of bias | “Thirty two adults participated in the current study” – “SBF data without motion artifacts were collected, extracted, and analyzed in 128 recording sessions” |
| Selective reporting (reporting bias) | - Unclear risk of bias | Insufficient information |
| Other bias :  Blinding of subjects to apparatus display | - Low risk of bias | Comment: probably blinded (picture) |
| Other bias :  Blinding of therapist to apparatus display | - Low risk of bias | Comment : There was a feedback to control load and frequency |
| Other bias  Blinding of the data extraction / cleaning process | - Unclear risk of bias | Insufficient information |
| Other bias  Blinding of the statistician | - Unclear risk of bias | Insufficient information |

| **Risk of Bias tool: Yung 2017** | | |
| --- | --- | --- |
| **Bias** | **Authors' judgement** | **Support for judgement** |
| Random sequence generation (selection bias) | - Low risk of bias | “the participants were randomly allocated by two assistants investigators at each university to either one of the two groups using a compuer-generated number system” |
| Allocation concealment (selection bias) | - Low risk of bias | “this allocation was concealed in an envelope” |
| Blinding of participants and personnel (performance bias) | - Unclear risk of bias | Comment : not tested with a questionnaire |
| Blinding of outcome assessment (detection bias) | - Unclear risk of bias | Insufficient information |
| Incomplete outcome data (attrition bias) | - Low risk of bias | Comment : 44 randomized, 44 analyzed |
| Selective reporting (reporting bias) | - Low risk of bias | Comment : protocol available and checked. No selective reporting |
| Other bias :  Blinding of subjects to apparatus display | - Low risk of bias | “throughout the entire data-collection process both the participants and therapists providing the intervention were blinded to the cardiovascular outcomes assessment” |
| Other bias :  Blinding of therapist to apparatus display | - Low risk of bias | “throughout the entire data-collection process both the participants and therapists providing the intervention were blinded to the cardiovascular outcomes assessment” |
| Other bias  Blinding of the data extraction / cleaning process | - Irrelevant | Irrelevant : no data extraction |
| Other bias  Blinding of the statistician | - Unclear risk of bias | Insufficient information |

| **Risk of Bias tool: Araujo 2017** | | |
| --- | --- | --- |
| **Bias** | **Authors' judgement** | **Support for judgement** |
| Random sequence generation (selection bias) | - Low risk of bias | “participants were randomly allocated to one of the three experimental groups” – “a blinded research assistant performed the allocation using a computerized based list…” |
| Allocation concealment (selection bias) | - Low risk of bias | “…and opaque sealed envelopes ensuring that the allocation was concealed” |
| Blinding of participants and personnel (performance bias) | - Low risk of bias | “seventy nine percent of the subject were unable to differentiate the real treatment from the placebo, and none of the individuals assigned to the placebo group could identify the placebo intervention as a placebo” |
| Blinding of outcome assessment (detection bias) | - Low risk of bias | “the outcome evaluator was blinded to group assignement” |
| Incomplete outcome data (attrition bias) | - Low risk of bias | Randomized : 60  Analyzed : 57 with reasons for exclusion provided.  Comment : Exclusions do not threat the validity of the results |
| Selective reporting (reporting bias) | - Low risk of bias | Comment : protocol available and checked. No selective reporting |
| Other bias :  Blinding of subjects to apparatus display | - Low risk of bias | “Participants and therapist were blinded to the outcome assessment” |
| Other bias :  Blinding of therapist to apparatus display | - Low risk of bias | “Participants and therapist were blinded to the outcome assessment” |
| Blinding of the data extraction / cleaning process | - Low risk of bias | “the 5 min sample with the most homogeneous signal was selected through visual inspection by a researcher blinded to group allocation” |
| Blinding of the statistician | - Unclear risk of bias | Insufficient information |

| **Risk of Bias tool: Henderson 2010** | | |
| --- | --- | --- |
| **Bias** | **Authors' judgement** | **Support for judgement** |
| Random sequence generation (selection bias) | - Unclear risk of bias | “randomly assigned” |
| Allocation concealment (selection bias) | - Unclear risk of bias | Insufficient information |
| Blinding of participants and personnel (performance bias) | - High risk of bias | “Only one participant who received placebo thought he or she had received OMT” |
| Blinding of outcome assessment (detection bias) | - Low risk of bias | Comment: (salivary and blood samples) |
| Incomplete outcome data (attrition bias) | - Unclear risk of bias | Comment: 3 participants excluded from the final analysis, their data “were used to refinement of the protocol” – “analyse limited by the small number of participant” |
| Selective reporting (reporting bias) | - Unclear risk of bias | Insufficient information |
| Other bias :  Blinding of subjects to apparatus display | Irrelevant | Irrelevant |
| Other bias :  Blinding of therapist to apparatus display | Irrelevant | Irrelevant |
| Blinding of the data extraction / cleaning process | - Low risk of bias | Comment: Probably equivalent |
| Blinding of the statistician | - Unclear risk of bias | Insufficient information |

| **Risk of Bias tool: Paungmali 2003** | | |
| --- | --- | --- |
| **Bias** | **Authors' judgement** | **Support for judgement** |
| Random sequence generation (selection bias) | - Low risk of bias | “concealed randomization (drawing lots)” |
| Allocation concealment (selection bias) | - Low risk of bias | Comment: probably done |
| Blinding of participants and personnel (performance bias) | - Unclear risk of bias | Comment: not tested with a questionnaire |
| Blinding of outcome assessment (detection bias) | - Low risk of bias | “The investigator responsible for collecting the data was unaware of the applied treatment condition” |
| Incomplete outcome data (attrition bias) | - Low risk of bias | “all 24 participants completed the study” |
| Selective reporting (reporting bias) | - Unclear risk of bias | Insufficient information |
| Other bias :  Blinding of subjects to apparatus display | - Low risk of bias | “Participants were unaware of the outcome measures”  Comment: Benefit of the doubt |
| Other bias :  Blinding of therapist to apparatus display | - Unclear risk of bias | Insufficient information |
| Blinding of the data extraction / cleaning process | - Unclear risk of bias | Insufficient information |
| Blinding of the statistician | - Unclear risk of bias | Insufficient information |

| **Risk of Bias tool: Moulson 2006** | | |
| --- | --- | --- |
| **Bias** | **Authors' judgement** | **Support for judgement** |
| Random sequence generation (selection bias) | - Unclear risk of bias | “the order of intervention was randomized for each participant” |
| Allocation concealment (selection bias) | - Unclear risk of bias | Insufficient information |
| Blinding of participants and personnel (performance bias) | - Low risk of bias | “No subject correctly guessed the purpose of the study”  Comment: Benefit of the doubt |
| Blinding of outcome assessment (detection bias) | - Low risk of bias | Comment: automated measure |
| Incomplete outcome data (attrition bias) | - Low risk of bias | Comment the 16 subjects provided post experimental questionnaire |
| Selective reporting (reporting bias) | - Unclear risk of bias | Insufficient information |
| Other bias :  Blinding of subjects to apparatus display | - Low risk of bias | “the recording monitor was not visible to the researcher or subject during the application of the intervention” |
| Other bias :  Blinding of therapist to apparatus display | - Low risk of bias | “the recording monitor was not visible to the researcher or subject during the application of the intervention” |
| Blinding of the data extraction / cleaning process | - Unclear risk of bias | Insufficient information |
| Blinding of the statistician | - Unclear risk of bias | Insufficient information |

| **Risk of Bias tool: Moutzouri 2012** | | |
| --- | --- | --- |
| **Bias** | **Authors' judgement** | **Support for judgement** |
| Random sequence generation (selection bias) | - Low risk of bias | “randomization was performed via a computer generated random number system” |
| Allocation concealment (selection bias) | - Unclear risk of bias | Insufficient information |
| Blinding of participants and personnel (performance bias) | - Unclear risk of bias | Comment: Assessed but results nor reported |
| Blinding of outcome assessment (detection bias) | - Low risk of bias | Automated measures |
| Incomplete outcome data (attrition bias) | - Low risk of bias | 45 randomized, 45 analyzed |
| Selective reporting (reporting bias) | - Unclear risk of bias | Insufficient information |
| Other bias :  Blinding of subjects to apparatus display | - Low risk of bias | “positioning ensured that neither the researcher nor the participants could receive any visual feedback” |
| Other bias :  Blinding of therapist to apparatus display | - Low risk of bias | “positioning ensured that neither the researcher nor the participants could receive any visual feedback” |
| Blinding of the data extraction / cleaning process | - Unclear risk of bias | Insufficient information |
| Blinding of the statistician | - Unclear risk of bias | Insufficient information |

| **Risk of Bias tool: Tsirakis 2015** | | |
| --- | --- | --- |
| **Bias** | **Authors' judgement** | **Support for judgement** |
| Random sequence generation (selection bias) | - Low risk of bias | “was randomly allocated to either the control, placebo or treatment group using the third party concealed randomization method” |
| Allocation concealment (selection bias) | - Low risk of bias | “sequentially numbered sealed envelopes” |
| Blinding of participants and personnel (performance bias) | - Low risk of bias | “no statistically significant difference in the perception of the participants as to whether they had received the treatment or the placebo condition” |
| Blinding of outcome assessment (detection bias) | - Low risk of bias | Automated measures |
| Incomplete outcome data (attrition bias) | - Low risk of bias | 45 randomized, 45 analyzed |
| Selective reporting (reporting bias) | - Unclear risk of bias | Insufficient information |
| Other bias :  Blinding of subjects to apparatus display | - Low risk of bias | “both treating therapist and the participant remained blind to SNS responses during the experimental period” |
| Other bias :  Blinding of therapist to apparatus display | - Low risk of bias | “both treating therapist and the participant remained blind to SNS responses during the experimental period” |
| Blinding of the data extraction / cleaning process | - Unclear risk of bias | Insufficient information |
| Blinding of the statistician | - Unclear risk of bias | Insufficient information |

| **Risk of Bias tool: Bowler 2017** | | |
| --- | --- | --- |
| **Bias** | **Authors' judgement** | **Support for judgement** |
| Random sequence generation (selection bias) | - Low risk of bias | “was randomized using computer generated random orders” |
| Allocation concealment (selection bias) | - Unclear risk of bias | Insufficient information |
| Blinding of participants and personnel (performance bias) | - Unclear risk of bias | Not tested with a questionnaire |
| Blinding of outcome assessment (detection bias) | - Low risk of bias | Automated measures |
| Incomplete outcome data (attrition bias) | - Low risk of bias | “all participants were included in the analysis” |
| Selective reporting (reporting bias) | - Unclear risk of bias | Insufficient information |
| Other bias :  Blinding of subjects to apparatus display | - Unclear risk of bias | Insufficient information |
| Other bias :  Blinding of therapist to apparatus display | - Unclear risk of bias | Insufficient information |
| Blinding of the data extraction / cleaning process | - Unclear risk of bias | Insufficient information |
| Blinding of the statistician | - Unclear risk of bias | Insufficient information |

| **Risk of Bias tool: Budgell 2001** | | |
| --- | --- | --- |
| **Bias** | **Authors' judgement** | **Support for judgement** |
| Random sequence generation (selection bias) | - Low risk of bias | “the order of the presentation of the authentic and sham manipulation was determined by a coin toss immediate prior to the first trial” |
| Allocation concealment (selection bias) | - Unclear risk of bias | Insufficient information |
| Blinding of participants and personnel (performance bias) | - High risk of bias | “hence the subjects were not generally blinded to whether they were receiving the authentic or the sham manipulation” |
| Blinding of outcome assessment (detection bias) | - Low risk of bias | Automated measures |
| Incomplete outcome data (attrition bias) | - Low risk of bias | One subject excluded after randomization. 25 randomized, 24 analyzed. |
| Selective reporting (reporting bias) | - Unclear risk of bias | Insufficient information |
| Other bias :  Blinding of subjects to apparatus display | - Unclear risk of bias | Insufficient information |
| Other bias :  Blinding of therapist to apparatus display | - Irrelevant | Irrelevant |
| Blinding of the data extraction / cleaning process | - Unclear risk of bias | Insufficient information |
| Blinding of the statistician | - Unclear risk of bias | Insufficient information |

| **Risk of Bias tool: Budgell 2006** | | |
| --- | --- | --- |
| **Bias** | **Authors' judgement** | **Support for judgement** |
| Random sequence generation (selection bias) | - Low risk of bias | “the order of presentation of the thoracic and sham manipulations was determined by a coin toss immediately before the first trial for each subject” |
| Allocation concealment (selection bias) | - Unclear risk of bias | Insufficient information |
| Blinding of participants and personnel (performance bias) | - High risk of bias | “In 3 of 31 trials, subjects incorrectly guessed that they had received thoracic manipulation on the occasion they had received a sham manipulation. One subject was unsure, and the remaining 27 subjects correctly guessed when they had received the sham procedure” |
| Blinding of outcome assessment (detection bias) | - Low risk of bias | Automated measures |
| Incomplete outcome data (attrition bias) | - Low risk of bias | 31 randomized, 28 analyzed |
| Selective reporting (reporting bias) | - Unclear risk of bias | Insufficient information |
| Other bias :  Blinding of subjects to apparatus display | - Unclear risk of bias | Insufficient information |
| Other bias :  Blinding of therapist to apparatus display | - Irrelevant | Irrelevant |
| Blinding of the data extraction / cleaning process | - Unclear risk of bias | Insufficient information |
| Blinding of the statistician | - Unclear risk of bias | Insufficient information |

| **Risk of Bias tool: Roy 2009** | | |
| --- | --- | --- |
| **Bias** | **Authors' judgement** | **Support for judgement** |
| Random sequence generation (selection bias) | - Low risk of bias | “the randomization was done by having each subject pick a number from a closed envelop” |
| Allocation concealment (selection bias) | - Unclear risk of bias | “the randomization was done by having each subject pick a number from a closed envelop”  Comment: Insufficient information |
| Blinding of participants and personnel (performance bias) | - Unclear risk of bias | Not assessed with a questionnaire |
| Blinding of outcome assessment (detection bias) | - Low risk of bias | Automated measures |
| Incomplete outcome data (attrition bias) | - Low risk of bias | 10 randomized, 10 analyzed  Comment: benefit of the doubt |
| Selective reporting (reporting bias) | - Low risk of bias | Protocole available and checked |
| Other bias :  Blinding of subjects to apparatus display | - Low risk of bias | Equivalent (watch) |
| Other bias :  Blinding of therapist to apparatus display | - Irrelevant | irrelevant |
| Blinding of the data extraction / cleaning process | - Unclear risk of bias | Insufficient information |
| Blinding of the statistician | - Unclear risk of bias | Insufficient information |

| **Risk of Bias tool: Sillevis 2010** | | |
| --- | --- | --- |
| **Bias** | **Authors' judgement** | **Support for judgement** |
| Random sequence generation (selection bias) | - Low risk of bias | “concealed allocation was performed by using a computer generated randomized table of numbers created before the beginning of the study.” |
| Allocation concealment (selection bias) | - Low risk of bias | “Individual, sequentially numbered index cards with random assignement were prepared and placed in sealed opaque envelopes.” |
| Blinding of participants and personnel (performance bias) | - Unclear risk of bias | Not tested with a questionnaire |
| Blinding of outcome assessment (detection bias) | - Low risk of bias | Automated measures |
| Incomplete outcome data (attrition bias) | - Low risk of bias | 101 randomized, 100 analyzed |
| Selective reporting (reporting bias) | - Unclear risk of bias | Insufficient information |
| Other bias :  Blinding of subjects to apparatus display | - Irrelevant | irrelevant |
| Other bias :  Blinding of therapist to apparatus display | - Irrelevant | irrelevant |
| Blinding of the data extraction / cleaning process | - Unclear risk of bias | Insufficient information |
| Blinding of the statistician | - Unclear risk of bias | Insufficient information |

| **Risk of Bias tool: Puhl 2012** | | |
| --- | --- | --- |
| **Bias** | **Authors' judgement** | **Support for judgement** |
| Random sequence generation (selection bias) | - Low risk of bias | “participants were randomly assigned to either the treatment or control group using a lottery method” |
| Allocation concealment (selection bias) | - Unclear risk of bias | Insufficient information |
| Blinding of participants and personnel (performance bias) | - Unclear risk of bias | Not tested with a questionnaire |
| Blinding of outcome assessment (detection bias) | - Low risk of bias | “nurse blinded to the participant’s allocation” |
| Incomplete outcome data (attrition bias) | - Low risk of bias | 56 randomized, 36 analyzed  Comment: most of the subjects were excluded before the intervention because of issue with the catether insertion” |
| Selective reporting (reporting bias) | - Unclear risk of bias | Insufficient information |
| Other bias :  Blinding of subjects to apparatus display | - Irrelevant | Irrelevant |
| Other bias :  Blinding of therapist to apparatus display | - Irrelevant | Irrelevant |
| Blinding of the data extraction / cleaning process | - Low risk of bias | “this allowed blinding of the laboratory investigator to the source of each blood sample” |
| Blinding of the statistician | - Unclear risk of bias | Insufficient information |

| **Risk of Bias tool: Ward 2013** | | |
| --- | --- | --- |
| **Bias** | **Authors' judgement** | **Support for judgement** |
| Random sequence generation (selection bias) | - Low risk of bias | “group assignment was determined by drawing random slips of paper” |
| Allocation concealment (selection bias) | - Unclear risk of bias | Insuffcient information |
| Blinding of participants and personnel (performance bias) | - Unclear risk of bias | Not tested with a questionnaire |
| Blinding of outcome assessment (detection bias) | - Low risk of bias | “the trained graduate research assistant takink all cardiovascular outcome measurements was however blinded to the study participant’s group” |
| Incomplete outcome data (attrition bias) | - Low risk of bias | 36 randomized, 36 analyzed |
| Selective reporting (reporting bias) | - Unclear risk of bias | Insufficient information |
| Other bias :  Blinding of subjects to apparatus display | - Unclear risk of bias | Insufficient information |
| Other bias :  Blinding of therapist to apparatus display | - Irrelevant | Irrelevant |
| Blinding of the data extraction / cleaning process | - Irrelevant | Irrelevant  Comment: no data extraction / cleaning needed |
| Blinding of the statistician | - Unclear risk of bias | Insufficient information |

| **Risk of Bias tool : Sampath 2017** | | |
| --- | --- | --- |
| **Bias** | **Authors' judgement** | **Support for judgement** |
| Random sequence generation (selection bias) | - Low risk of bias | “the randomization schedule was prepared by a research administrator using acomputer generated random numbers table” |
| Allocation concealment (selection bias) | - Low risk of bias | “This was done by the use of sealed opaque envelopes” |
| Blinding of participants and personnel (performance bias) | - Unclear risk of bias | Not tested with a questionnaire |
| Blinding of outcome assessment (detection bias) | - Low risk of bias | “the outcome assessor…were blinded” |
| Incomplete outcome data (attrition bias) | - Low risk of bias | “there was no missing data”  24 randomized, 24 analyzed |
| Selective reporting (reporting bias) | - Unclear risk of bias | Insufficient information |
| Other bias :  Blinding of subjects to apparatus display | - Unclear risk of bias | Insufficient information |
| Other bias :  Blinding of therapist to apparatus display | - Irrelevant | Irrelevant |
| Blinding of the data extraction / cleaning process | - Unclear risk of bias | Insufficient information |
| Blinding of the statistician | - Unclear risk of bias | Insufficient information |
