# Supplementary material for: The acute effects of joint manipulative techniques on markers of autonomic nervous system activity: a systematic review and meta-analysis of randomized sham-controlled trials
Source: Chiropr Man Therap. 2019 Mar 12;27:17. doi: 10.1186/s12998-019-0235-1 (PMC6413458; doi:10.1186/s12998-019-0235-1)
Supplement: Supplementary file 3 — Technical quality check-list. This file contains the technical quality check-list (scoring system, summary, and details). (DOCX 43 kb) [file 12998_2019_235_MOESM3_ESM.docx]

**Additional file 3**

**Technical quality check-list**

| **Technical quality check-list (scoring system)** | | | | | | | | |
| --- | --- | --- | --- | --- | --- | --- | --- | --- |
| **Study ID** | **Treatment or intervention performed by**  **experienced person?**  **/1** | **Interventions & control procedures described?**  **/1** | **1.Was main outcome measure reported as reproducible, reliable or was it tested in study?**  **2.And if so, was reference correct or did own test show acceptable results?**  **/1** | **Data acquisition**  **1. Controlled experimental conditions?**  **2.Was the measurement procedure described?**  **3. Was the duration of the stabilizing period acceptable?**  **4. Were measurements performed by experienced person?**  **5. Adequate sampling rate?**    **/5** | **Data cleaning process described?**  **/1** | **Was power calculation done?**  **/1** | **Score** | **‘Mechanical’ profile of the sham procedure** |
|  | **yes (1 point)**  **no or ‘?’ (0 point)** | **yes (1 point)**  **no (0 point)** | **1.**  **yes (0.5 point)**  **no (0 point)**  **2.**  **yes (0.5 point)**  Providing at least the ICC score or equivalent  **no (0 point)** | **1.**  Did they control one or several of the following parameters which may have an impact on autonomic measures, e.g. temperature, humidity, food / caffeine / tobacco / alcohol intakes, physical activity, breathing rate?  **yes (1 point)**  **no or ‘?’ (0 point)**  **2**.  **yes (1 point)**  **no (0 point)**  **3.**  **yes ≥ 5 min (1 point)**  **no < 5 min or ‘?’ (0 point)**  **4.**  **yes (1 point)**  **no or ‘?’ (0 point)**  **5.**  **yes (1 point)**  For SC, ST, SBF: at least 20 Hz, 100-200 Hz is better  For HRV: 1000 Hz or higher values  **no or ‘?’ (0 point)** | Did they provide information about the visual analysis of the raw data (e.g. ECG, SC, ST, SBF)?  Did they provide information about how they dealt with measurement issues (e.g. artifact correction)?  **yes (1 point)**  **no or ‘?’ (0 point)** | **yes (1 point)**  If the power calculation was performed for the primary autonomic outcome variable  **no (0 point)**  **Not applicable**  If power calculation was not performed but there was a statistically significant difference between the JMT and the Sham for at least one of the main outcome variables.  Or  If it is a pilot / preliminary study  Or  If the power calculation was based on another variable |  | **1. Similar to the JMT**  e.g. sham mobilization performed with oscillatory movements (without the joint component)  e.g. sham SNAGs performed with a sustained pressure (without the joint component)  e.g. sham HVLA manipulation performed with preload and thrust phases (without the joint component)  **2. Manual contact without movement**  **3. Activator (instrument)** |

| **Technical quality Check-list. Summary**  **Mobilizations (oscillatory technique)** | | | | | | | | |
| --- | --- | --- | --- | --- | --- | --- | --- | --- |
| **Study ID** | **Treatment or intervention performed by**  **experienced person?** | **Interventions & control procedures described?** | **1.Was main outcome measure reported as reproducible, reliable or was it tested in study?**  **2.And if so, was reference correct or did own test show acceptable results?** | **Data acquisition**  **1. Controlled experimental conditions?**  **2.Was the measurement procedure described?**  **3. Was the duration of the stabilizing period acceptable?**  **4. Were measurements performed by experienced person?**  **5. Adequate sampling rate?** | **Data cleaning process described?** | **Was power calculation done?** | **Score** | **‘Mechanical’ profile of the sham procedure** |
| Petersen 1993 [35] | 1 | 1 | 0 | 3/4 | 0 | 1 | 66 % | Manual contact |
| Vicenzino 1994 [36] | ? | 1 | 0 | 4 | 0 | Not applicable (NA) | 55 % | Manual contact |
| Slater 1994 [37] | ? | 1 | 0 | 4 | 0 | NA | 55 % | Manual contact |
| Simon 1997 [38] | ? | 1 | 0 | 4 | 0 | NA | 55 % | Manual contact |
| McGuiness 1997 [39] | 1 | 1 | 0 | 3/4 | 0 | NA | 62 % | Manual contact |
| Vicenzino 1998 [40] | ? | 0 | 0 | 2/4 | 0 | NA | 25 % | Sham not described |
| Vicenzino 1998 [41] | ? | 1 | 0 | 3 | 0 | NA | 44 % | Manual contact |
| Sterling 2001 [42] | 1 | 1 | 0 | 3 | 0 | NA | 55 % | Manual contact |
| Perry 2008 [43] | ? | 1 | 0 | 4 | 0 | 1 | 60 % | Manual contact |
| Jowsey 2010 [44] | 1 | 1 | 1/2 | 3 | 0 | 1 | 65 % | Manual contact |
| La Touche 2013 [45] | 1 | 1 | 0 | 4 | 0 | NA | 66 % | Manual contact |
| Yung 2014 [46] | ? | 1 | 1 | 3/4 | 0 | 1 | 66 % | Manual contact |
| Piekarz 2016 [47] | 1 | 1 | 1 | 4 | 1 | 1 | 90 % | Manual contact |
| Zegarra 2016 [48] | 1 | 1 | 0 | 4 | 1 | 1 | 80 % | Similar with less pressure |
| Yung 2017 [49] | 1 | 1 | 1 | 3/4 | 0 | 1 | 77 % | Manual contact |
| Araujo 2017 [50] | 1 | 1 | 1/2 | 4/5 | 1 | 1 | 85 % | Manual contact |
| **Mobilizations (atypical technique)** | | | | | | | | |
| Henderson 2010 [51] | 1 | 1 | 1 | 3/4 | 1 | 1 | 88 % | Manual contact |

| **Technical quality Check-list. Summary**  **Mobilizations (SNAGs / mobilization with movement)** | | | | | | | | |
| --- | --- | --- | --- | --- | --- | --- | --- | --- |
| **Study ID** | **Treatment or intervention performed by**  **experienced person?** | **Interventions & control procedures described?** | **1.Was main outcome measure reported as reproducible, reliable or was it tested in study?**  **2.And if so, was reference correct or does own test show acceptable results?** | **Data acquisition**  **1. Controlled experimental conditions?**  **2.Was the measurement procedure described?**  **3. Was the duration of the stabilizing period acceptable?**  **4. Are measurements performed by experienced person?**  **5. Adequate sampling rate?** | **Data cleaning process described?** | **Was power calculation done?** | **Score** | **‘Mechanical’ profile of the sham procedure** |
| Paungmali 2003 [52] | 1 | 1 | 1 | 3 | 0 | NA | 66 % | Manual contact |
| Moulson 2006 [53] | ? | 1 | 0 | 4 | 0 | NA | 55 % | Manual contact |
| Moutzouri 2012 [54] | 1 | 1 | 1/2 | 4 | 0 | NA | 72 % | Manual contact |
| Tsirakis 2015 [55] | 1 | 1 | 0 | 2 | 0 | 1 | 50 % | Manual contact |
| Bowler 2017 [56] | 1 | 1 | 1 | 3 | 0 | 1 | 70 % | Manual contact |
| **HVLA manipulation** | | | | | | | | |
| Budgell 2001 [57] | 1 | 1 | 0 | 1 | 1 | 0 | 40 % | Similar to the JMT |
| Budgell 2006 [58] | ? | 1 | 0 | 2 | 1 | 1 | 50 % | Similar to the JMT |
| Roy 2009 [59] | 1 | 1 | 0 | 1 | 0 | 1 | 40 % | Manual contact |
| Sillevis 2010 [60] | 1 | 1 | 1 | 3 | 0 | 0 | 60 % | Manual contact |
| Puhl 2012 [61] | 1 | 1 | 0 | 4/4 | 1 | 0 | 77 % | Manual contact |
| Ward 2013 [62] | 1 | 1 | 0 | 4/4 | 0 | 0 | 66 % | Activator |
| Sampath 2017 [63] | 1 | 1 | 1 | 3 | 0 | NA | 66 % | Manual contact |

| **Technical quality Check-list with details**  **Mobilizations (oscillatory technique)** | | | | | | | | |
| --- | --- | --- | --- | --- | --- | --- | --- | --- |
| **Study ID** | **Treatment or intervention performed by**  **experienced person?** | **Interventions & control procedures described?** | **1.Was main outcome measure reported as reproducible, reliable or was it tested in study?**  **2.And if so, was reference correct or did own test show acceptable results?** | **Data acquisition**  **1. Controlled experimental conditions?**  **2.Was the measurement procedure described?**  **3. Was the duration of the stabilizing period acceptable?**  **4. Were measurements performed by experienced person?**  **5. Adequate sampling rate?** | **Data cleaning process described?** | **Was power calculation done?** | **Score** | **‘Mechanical’ profile of the sham procedure** |
| Petersen  1993  [35] | yes  manipulative therapist | yes | no | 1. yes (food, drink, caffeine, nicotine, temperature, humidity)  2. yes  3. yes (10 min)  4.?  5.not relevant | no | yes | 6/9 | Manual contact without movement |
| Vicenzino  1994  [36] | ? | yes | no | 1. yes (physical activity, caffeine, nicotine, temperature, humidity, sound)  2. yes  3. yes (10 min)  4.?  5. yes (20 Hz) | no | Not applicable | 5/9 | Manual contact without movement |
| Slater  1994  [37] | ? | yes | no | 1. yes (physical activity, alcohol caffeine, nicotine, temperature,)  2. yes  3. yes (10 min)  4.?  5. yes (20 Hz) | no | Not applicable | 5/9 | Manual contact without movement |
| Simon  1997  [38] | ? | yes | no | 1. yes (caffeine, temperature, humidity, sound)  2. yes  3.yes (10 min)  4?  5. yes (20 Hz) | no | Not applicable  Preliminary study | 5/9 | Manual contact without movement |

| **Technical quality Check-list with details**  **Mobilizations (oscillatory technique)** | | | | | | | | |
| --- | --- | --- | --- | --- | --- | --- | --- | --- |
| **Study ID** | **Treatment or intervention performed by**  **experienced person?** | **Interventions & control procedures described?** | **1.Was main outcome measure reported as reproducible, reliable or was it tested in study?**  **2.And if so, was reference correct or did own test show acceptable results?** | **Data acquisition**  **1. Controlled experimental conditions?**  **2.Was the measurement procedure described?**  **3. Was the duration of the stabilizing period acceptable?**  **4. Were measurements performed by experienced person?**  **5. Adequate sampling rate?** | **Data cleaning process described?** | **Was power calculation done?** | **Score** | **‘Mechanical’ profile of the sham procedure** |
| McGuiness  1997  [39] | yes  physiotherapist | yes | no | 1. yes (temperature, humidity, noises, alcohol, caffeine, exercises)  2. yes  3 yes. (5 min)  4?  5. not relevant | no | Not applicable | 5/8 | Manual contact without movement |
| Vicenzino 1998  [40] | ? | Intervention: yes  Placebo, control: no | no | 1. yes (noise, temperature)  2. yes  3.?  4.?  5. Not relevant | no | Not applicable | 2/8 | Sham not described |
| Vicenzino  1998  [41] | ? | yes | no | 1. yes  2. yes  3.?  4.?  5. yes (20 Hz) | no | Not applicable | 4/9 | Manual contact without movement |
| Sterling  2001  [42] | yes  physiotherapist | yes | no | 1. yes (noise, temperature, humidity)  2. yes  3.?  4.?  5. yes (20 Hz) | no | Not applicable | 5/9 | Manual contact without movement |

| **Technical quality Check-list with details**  **Mobilizations (oscillatory technique)** | | | | | | | | |
| --- | --- | --- | --- | --- | --- | --- | --- | --- |
| **Study ID** | **Treatment or intervention performed by**  **experienced person?** | **Interventions & control procedures described?** | **1.Was main outcome measure reported as reproducible, reliable or was it tested in study?**  **2.And if so, was reference correct or did own test show acceptable results?** | **Data acquisition**  **1. Controlled experimental conditions?**  **2.Was the measurement procedure described?**  **3. Was the duration of the stabilizing period acceptable?**  **4. Were measurements performed by experienced person?**  **5. Adequate sampling rate?** | **Data cleaning process described?** | **Was power calculation done?** | **Score** | **‘Mechanical’ profile of the sham procedure** |
| Perry  2008  [43] | ? | yes | no | 1. yes (temperature)  2. yes  3. yes (10 min)  4.?  5. yes (20 Hz) | no | yes | 6/10 | Manual contact without movement |
| Jowsey  2010  [44] | yes  physiotherapist | yes | 1. yes  2. no | 1. yes (temperature)  2. yes  3.yes (8 min)  4.?  5.? | no | yes | 6.5/10 | Manual contact without movement |
| La Touche  2013  [45] | yes  physiotherapist | yes | no | 1. yes (temperature)  2. yes  3. yes (10 min)  4. yes  5.? | no | Not applicable,  power calculation was performed for the PPT | 6/9 | Manual contact without movement |
| Yung  2014  [46] | ? | yes | 1.yes  1. yes  ICC for HR: 0.97  ICC for SBP: 0.98  ICC for DBP: 0.88 | 1. yes (caffeine, alcohol, exercise)  2. yes  3. yes (5 min)  4.?  5. Not relevant | no | yes | 6/9 | Manual contact without movement |

| **Technical quality Check-list with details**  **Mobilizations (oscillatory technique)** | | | | | | | | |
| --- | --- | --- | --- | --- | --- | --- | --- | --- |
| **Study ID** | **Treatment or intervention performed by**  **experienced person?** | **Interventions & control procedures described?** | **1.Was main outcome measure reported as reproducible, reliable or was it tested in study?**  **2.And if so, was reference correct or did own test show acceptable results?** | **Data acquisition**  **1. Controlled experimental conditions?**  **2.Was the measurement procedure described?**  **3. Was the duration of the stabilizing period acceptable?**  **4. Were measurements performed by experienced person?**  **5. Adequate sampling rate?** | **Data cleaning process described?** | **Was power calculation done?** | **Score** | **‘Mechanical’ profile of the sham procedure** |
| Piekarz  2016  [47] | yes  physiotherapist | yes | 1. yes  2. yes  ICC: 0,997 | 1. yes (temperature, caffeine, alcohol, nicotine, strenuous activity)  2. yes  3.yes (8 min)  4.?  5. yes (200 Hz) | yes (remained at the discretion of the data assessor) | yes | 9/10 | Manual contact without movement |
| Zegarra-parodi  2016  [48] | yes | yes | no | 1. yes (caffeine, alcohol, strenuous exercise, breath rate)  2. yes  3. yes (20 min)  4.?  5. yes (1000 Hz) | yes (SBF data without motion artifact were collected, extracted, and analyzed) | yes | 8/10 | Similar to the JMT with less pressure |
| Yung  2017  [49] | yes | yes | Yes (previous study) | 1. yes (caffeine, alcohol, exercise)  2. yes  3. yes (5min)  4.?  5. Not relevant | no | yes | 7/9 | Manual contact without movement |
| Araujo  2017  [50] | yes | yes | 1. yes  2. no (good agreement between Polar and ECG) | 1. yes  2. yes  3. yes (5min)  4.?  5. yes (1000 Hz) | yes | yes | 8.5/10 | Manual contact without movement |

| **Technical quality Check-list with details**  **Mobilizations (atypical technique)** | | | | | | | | |
| --- | --- | --- | --- | --- | --- | --- | --- | --- |
| Henderson 2010 [51] | yes  osteopath | yes | 1. yes  2. yes | 1. yes (excluded if they consume anything other than water within an hour of the appointment)  2.yes  3.?  4.yes  5.Not relevant | yes | yes (3) | 8/9 | Manual contact without movement |

| **Technical quality Check-list with details**  **Mobilizations (SNAGs / mobilization with movement)** | | | | | | | | |
| --- | --- | --- | --- | --- | --- | --- | --- | --- |
| **Study ID** | **Treatment or intervention performed by**  **experienced person?** | **Interventions & control procedures described?** | **1.Was main outcome measure reported as reproducible, reliable or was it tested in study?**  **2.And if so, was reference correct or did own test show acceptable results?** | **Data acquisition**  **1. Controlled experimental conditions?**  **2.Was the measurement procedure described?**  **3. Was the duration of the stabilizing period acceptable?**  **4. Were measurements performed by experienced person?**  **5. Adequate sampling rate?** | **Data cleaning process described?** | **Was power calculation done?** | **Score** | **‘Mechanical’ profile of the sham procedure** |
| Paungmali  2003  Australia  [52] | yes  physical  therapist | yes | 1. yes  2. yes | 1. yes (temperature, humidity, noise, nicotine, caffeine, nicotine)  2. yes  3.?  4.?  5. yes (20 Hz) | no | Not applicable  power calculation based on the PPT | 6/9 | Manual contact without movement |
| Moulson  2006  UK  [53] | ? | yes | no “accepted as an indirect measure of SNS” | 1. yes (temperature, humidity, noise, nicotine, caffeine, strenuous exercises)  2. yes  3. yes (8 min)  4.?  5. yes (50 Hz) | no | Not applicable  preliminary study | 5/9 | Manual contact without movement |
| Moutzouri  2012  UK  [54] | yes  physiotherapist | yes | 1. yes  2. no | 1. yes (temperature, sound, exercises, caffeine, nicotine, alcohol)  2. yes  3. yes (8 min)  4.?  5. yes (20 Hz) | no | Not applicable  first study | 6.5/9 | Manual contact without movement |
| Tsirakis  2015  UK  [55] | yes  manual therapist | yes | no | 1.?  2. yes  3. yes (10 min)  4.?  5.? | no | yes | 5/10 | Manual contact without movement |
| Bowler  2017  UK [56] | yes  physiotherapist | yes | 1. yes  2. yes | 1. yes (temperature, exercises, caffeine, nicotine, alcohol)  2. yes  3. yes (8 min)  4.?  5.? | no | yes | 7/10 | Manual contact without movement |

| **Technical quality Check-list with details**  **HVLA manipulation** | | | | | | | | |
| --- | --- | --- | --- | --- | --- | --- | --- | --- |
| **Study ID** | **Treatment or intervention performed by**  **experienced person?** | **Interventions & control procedures described?** | **1.Was main outcome measure reported as reproducible, reliable or was it tested in study?**  **2.And if so, was reference correct or did own test show acceptable results?** | **Data acquisition**  **1. Controlled experimental conditions?**  **2.Was the measurement procedure described?**  **3. Was the duration of the stabilizing period acceptable?**  **4. Were measurements performed by experienced person?**  **5. Adequate sampling rate?** | **Data cleaning process described?** | **Was power calculation done?** | **Score** | **‘Mechanical’ profile of the sham procedure** |
| Budgell  2001  [57] | yes  chiropractor | yes | no | 1.?  2. yes  3.?  4.?  5.? | yes (benefit of the doubt) | no | 4/10 | Similar to the JMT  (without the joint component) |
| Budgell  2006  [58] | ? | yes | no | 1.yes breathing rate (0.25 Hz)  2. yes  3.?  4.?  5.? | yes, visual analysis performed | yes | 5/10 | Similar to the JMT  (without the joint component) |
| Roy  2009  Canada  [59] | yes  chiropractors | yes | no | 1.?  2. yes  3..no (3 min; 8-5)  4.?  5.? | no | yes (benefit of the doubt) | 4/10 | Manual contact without movement |
| Sillevis  2010  USA  [60] | yes  physiotherapist | yes | 1. yes  2. yes | 1. yes (caffeine, tobacco, food)  2. yes  3. probably (benefit of the doubt, 3 min in the dark)  4.?  5.? | no | no | 6/10 | Manual contact without movement |
| Puhl  2012  Canada  [61] | yes  chiropractor | yes | no | 1. yes (food, caffeine, tobacco)  2. yes  3. yes (10min)  4. yes (nurse)  5. not applicable | yes (benefit of the doubt) | no | 7/9 | Manual contact without movement |
| Ward  2013  USA  [62] | yes  chiropractor | yes | no | 1. yes (caffeine, alcohol, tobacco, temperature)  2. yes  3.yes (5 min)  4. yes  5. not relevant | no | no | 6/9 | Activator (no force) |
| Sampath  2017  New Zealand  [63] | yes  physical therapist | yes | 1. yes  2. yes  (for near infrared spectroscopy) | 1. yes (temperature, humidity)  2. yes  3. yes (5 min)  4.?  5.? | no | Not applicable | 6/9 | Manual contact without movement |
