# Supplementary material for: The acute effects of joint manipulative techniques on markers of autonomic nervous system activity: a systematic review and meta-analysis of randomized sham-controlled trials
Source: Chiropr Man Therap. 2019 Mar 12;27:17. doi: 10.1186/s12998-019-0235-1 (PMC6413458; doi:10.1186/s12998-019-0235-1)
Supplement: Supplementary file 4 — Results table. This file contains the results for each included study. (DOCX 40 kb) [file 12998_2019_235_MOESM4_ESM.docx]

**Additional file 4**

**Results table**

| **Results table** **Mobilizations (oscillatory technique)** | | | | | | | | | | | | | | | |
| --- | --- | --- | --- | --- | --- | --- | --- | --- | --- | --- | --- | --- | --- | --- | --- |
| **Study ID** | **Joint manipulative technique** | **Measurement period**  **(duration)** | **Outcomes** | **Statistical analysis: statistical tests,** **Level** | **Summary of the results**  **(Comparison between JMT and Sham)** | | | | | | | | | | **Other findings**  **and comments** |
| Petersen  1993 [35] | C5 Grade III postero-anterior mobilization (central pressure) | **-** Baseline (2 min)  - Intervention (5 min)  - Post intervention (5 min) | - SC  - ST  (% change from baseline) | - Repeated measures ANOVA (intervention factor)  - Post hoc analysis with Duncan’s multiple range test, Fisher’s protected least squares difference test  - Alpha: 0.05 | ***SC intervention period*** | | | | ***SC post intervention period*** | | | | ***ST intervention and***  ***post intervention periods*** | | JMT produced a 50-60 % increase in SC. The Sham produced an initial increase in SC of 30 % and after an increase of 15-20 %. Greatest difference during the treatment period.  No statistically significant difference between Sham and Control for both SC and ST |
|  |  |  |  |  | JMT produced a statistically significant increase in SC (after 45 sec), compared to Sham.  *p < 0.05* | | | | JMT produced a statistically significant increase in SC compared to Sham only during the first 30 sec. *p < 0.05* | | | | No statistically significant difference. | |  |
| Vicenzino  1994 [36] | 1. C5/C6 grade III left lateral glide mobilization + ULTT1  2. C5/C6 grade III left lateral glide mobilization + ULTT2 | - Baseline (2 min)  - Intervention (3min30)  - Post Intervention | - SC AUC  - SC MAX  - SC Tmax  - SC Thalf  - ST AUC  - ST MAX (MIN)  - ST Tmax  - ST Thalf  (% change from baseline) | - MANOVA (Side factor, intervention factor)  - A priori orthogonal contrasts  - Alpha: 0.05 | ***SC AUC intervention + post intervention periods*** | | | | ***SC MAX post intervention period*** | | | | ***SC: Tmax, Thalf***  ***ST: AUC, MIN, Tmax, Thalf*** | | No statistically significant difference between both techniques.  No side difference.  No statistically significant difference between Sham and Control for SC AUC and SC MAX |
|  |  |  |  |  | 1. JMT produced a statistically significant increase in SC AUC (23 %) compared to Sham (7 %).  p = 0.000 | | | | 1. JMT produced a statistically significant increase in SC MAX (97 %) compared to Sham (50 %).  p = 0.000 | | | | No statistically significant difference | |  |
|  |  |  |  |  | 2. JMT produced a statistically significant increase in SC AUC (33 %) compared to sham (7 %). *p = 0.000* | | | | 2. JMT produced a statistically significant increase in SC MAX (124 %) compared to sham (50 %). *p = 0.000* | | | | No statistically significant difference | |  |
| Slater  1994 [37] | T6 (right costo-vertebral joint) grade IV postero-anterior mobilization +  ” sympathetic slump” position | - Baseline (2 min)  - Intervention (3min)  - Post intervention (10 min) | - SC AUC  - SC MAX  - ST AUC  - ST MAX (MIN)  (% change from baseline) | -MANOVA (side factor, intervention factor)  - Post Hoc analysis with Duncan test  - Alpha: 0.05 | ***SC AUC intervention + post intervention periods*** | | | | ***SC MAX intervention + post intervention periods*** | | | | ***ST AUC, ST MAX(MIN)*** | | Sham produced a statistically significant increase in SC AUC and SC MAX compared to Control  Sham produced a statistically significant decrease in ST AUC compared to Control during the intervention period  No information about the effect in the post-treatment period alone |
|  |  |  |  |  | JMT produced a statistically significant increase in SC AUC compared to sham for both side (*p < 0.05*). The increase was significantly greater for the right side than for the left only during the intervention period. | | | | JMT produced a statistically significant increase in SC MAX (200 %) compared to sham (150 %) for both side (*p < 0.05*). The increase was significantly greater for the right side than for the left only during the intervention period. | | | | No statistically significant difference | |  |
| Simon  1997 [38] | Right glenohumeral joint grade III antero-posterior mobilization | -Baseline (2 min)  - Intervention (3min30)  -Post intervention (10 min) | - SC treatment AUC  - SC total AUC  - SC MAX  - ST treatment AUC  - ST total AUC  - ST MIN  (% change from baseline) | - ANOVA (side factor, intervention factor)  - A priori contrasts  - Alpha: | ***SC treatment AUC (intervention period)*** | ***SC total AUC (intervention + post intervention periods)*** | | | | **SC MAX** | | ***ST MIN*** | | ***ST AUC treatment period and ST Total AUC*** | No side difference for the SC  No information about the effect in the post treatment period alone. We can assume the effect decrease as the « SC AUC total » was lesser than the « SC AUC intervention » |
|  |  |  |  |  | JMT produced a statistically significant increase in SC AUC (≈ 150 %) compared to sham (≈ 100 %).  *p = 0.0022* | JMT produced a statistically significant increase in SC Total AUC (≈ 90%) compared to sham (≈ 50 %).  *p = 0.0001* | | | | JMT produced a statistically significant increase in SC MAX (≈ 250 %) compared to sham (≈ 140 %).  *p = 0.0001* | | JMT produced a statistically significant decrease in ST MIN for the right side (≈ - 5.5 %) compared to sham (≈ -4.5 %).  p = 0.0192 | | No statistically significant difference |  |
| SC AUC: skin conductance area under the curve ; SC MAX : skin conductance maximum value ; SC Tmax : skin conductance time taken to achieve the maximum effect ; SC Thalf : skin conductance time for the effect decrease to half ; ST AUC : skin temperature area under the curve ; ST MAX : skin temperature maximum value ; ST Tmax : skin temperature time taken to achieve the maximum effect ; ST Thalf : skin temperature time for the effect decrease to half ; ULTT : upper limb tension test | | | | | | | | | | | | | | | |
| **Results table**  **Mobilizations (oscillatory technique)** | | | | | | | | | | | | | | | |
| **Study ID** | **Joint manipulative technique** | **Measurement period**  **(duration)** | **Outcomes** | **Statistical analysis: statistical tests,** **Level** | **Summary of the results**  **(Comparison between JMT and Sham)** | | | | | | | | | | **Other findings**  **and comments** |
| McGuiness  1997 [39] | C5 Grade III postero-anterior mobilization (central) | - Baseline (4min)  - Intervention (5 min) | - RR MAX  - HR MAX  - Systolic and diastolic BP MAX  (% change from baseline) | - ANOVA  - A priori contrasts  - Alpha: | ***HR MAX*** | | | **RR MAX** | | | | | ***BP MAX*** | | Graphical analysis does not show a difference between Sham and Control excepted for RR (Control RR > Sham RR). |
|  |  |  |  |  | JMT produced a statistically significant increase in HR MAX (+10.5 %) compared to Sham (no change).  *p = 0.0012* | | | JMT produced a statistically significant increase in RR MAX (44%) compared to Sham (no change).  p = 0.0001 | | | | | JMT produced a statistically significant increase in diastolic BP (+12.5%) and in systolic BP (+4.5%) compared to Sham (no change).  *p* *= 0.0006 and*  *p = 0.0016* | |  |
| Vicenzino  1998 [40] | C5 Grade III left lateral glide mobilization + ULTT 2 | -Baseline (2 min)  - Intervention (3min30) | - HR MAX  - RR MAX  - BP MAX  (% change from baseline) | -Within-subjects a-priori contrasts (intervention factor)  -Alpha: 0.05 | ***HR MAX*** | | | ***RR MAX*** | | | | | ***BP MAX*** | | No difference between sham and control |
|  |  |  |  |  | JMT produced a statistically significant increase in HR MAX (13%) compared to sham (2 %).  *p = 0.001* | | | JMT produced a statistically significant increase in RR MAX (36%) compared to sham (13%).  *p = 0.000* | | | | | JMT produced a statistically significant increase in both diastolic and systolic BP (14%) compared to sham (1%).  *p = 0.000* | |  |
| Vicenzino  1998 [41] | C5/C6 Grade III oscillatory lateral glide mobilization | - Baseline (2 min)  - Intervention (3 min 30) | - SC MAX  - ST MIN  - SBF MAX  (% change from baseline) | - ANOVA (intervention factor)  - A priori contrasts (difference between interventions)  -Alpha: 0.05 | ***SC MAX*** | | ***ST MIN (hand)*** | | | | ***SBF MAX (hand and elbow)*** | | | **ST Elbow** |  |
|  |  |  |  |  | JMT produced a statistically significant increase in SC MAX (69.05 ± 13.70%) compared to sham (≈ 30%).  *p < 0.05* | | JMT produced a statistically significant decrease in Hand ST MIN (-3.45 ± 0.4%) compared to sham (≈ no change).  *p < 0.05* | | | | JMT produced a statistically significant increase in Elbow SBF (127.5 ± 27.8%) compared to sham (≈ no change). *p < 0.05*  JMT produced a statistically significant decrease in Hand SBF (-35.30 ± 5.1%) compared to sham (≈ -5%). *p < 0.05* | | | No statistically significant difference |  |
| Sterling  2001 [42] | C5/C6 symptomatic side Grade III postero-anterior mobilization | -Baseline (2 min)  -Intervention (6 min) | - SC AUC  - SC MAX  - ST AUC  - ST MIN  (% change from baseline) | - ANOVA (side factor, intervention factor)  - Post Hoc analysis with Newman-Keuls test  - Alpha: 0.05 | ***SC AUC*** | | ***SC MAX*** | | | | ***ST MIN*** | | | ***ST AUC*** | No side difference.  No difference between Sham and Control for SC AUC (figure analysis) |
|  |  |  |  |  | JMT produced a statistically significant increase in SC AUC (16 ± 2.96 %) compared to Sham (≈ 4%).  *p = 0.001* | | JMT produced a statistically significant increase in SC MAX (114 ± 10.5%) compared to Sham.  *p = 0.0003* | | | | JMT produced a statistically significant decrease in ST MIN (- 2.5 ± 0.5 %) compared to Sham.  *p = 0.022* | | | No statistically significant difference |  |
| BP MAX: blood pressure maximum effect; HR MAX: heart rate maximum effect; RR MAX: respiratory rate maximum effect; SBF: skin blood flow; SC: skin conductance; ST: skin temperature; MAX: maximum value; Min: minimum value; | | | | | | | | | | | | | | | |

| **Results table**  **Mobilizations (oscillatory technique**) | | | | | | | | | | | | |
| --- | --- | --- | --- | --- | --- | --- | --- | --- | --- | --- | --- | --- |
| **Study ID** | **Joint manipulative technique** | **Measurement period**  **(duration)** | **Outcomes** | **Statistical analysis: statistical tests,** **Level** | **Summary of the results**  **(Comparison between JMT and Sham)** | | | | | | | **Other findings**  **and comments** |
| Perry  2008 [43] | Left L4 / L5 facet joint Grade III oscillatory mobilization at 2 Hz | - Baseline (2 min)  - Intervention (5 min)  - Post intervention (5 min) | - SC Integral measurement  (% change from baseline) | - Multivariate analysis (side factor, intervention factor, time factor)  - Post Hoc analysis with Tukey’s HSD  - Alpha: 0.05 | ***SC integral measurement intervention*** | | | ***SC integral measurement post intervention*** | | | | No statistically significant difference between Sham and Control |
|  |  |  |  |  | JMT produced a statistically significant increase in SC in the left lower limb (13.47 %) compared to Sham (-1.93 %). *p < 0.05* | | | No statistically significant difference | | | |  |
| Jowsey  2010 [44] | T4 Grade III rotatory postero-anterior mobilization at 0.5 Hz | - Baseline (2 min)  - Intervention (5 min)  - Post intervention (5 min) | -SC Integral measurement  (% change from baseline) | - ANOVA (intervention factor)  - Alpha: 0.05 | ***SC intervention period*** | | | ***SC post intervention period*** | | | | There was a trend towards a bilateral effect during the post intervention period |
|  |  |  |  |  | No statistically significant difference | | | JMT produced a statistically significant increase in SC (4.47; 95 % CI [-8.54; 17.49]) in the right hand compared to Sham (- 12.38 %; 95 CI [ -21.62; -2.98]). *p = 0.034* | | | |  |
| La Touche  2013 [45] | C0-C3 Antero-posterior upper cervical mobilization  at 0.5 Hz | - Baseline  - Post Intervention | - SC  - ST  - HR  - BR | - ANOVA (intervention factor; time factor)  - Post Hoc analysis with Bonferroni corrections  - Alpha: 0.05 | ***SC*** | ***BR*** | | | ***HR*** | | ***ST*** |  |
|  |  |  |  |  | JMT produced a statistically significant increase in SC compared to Sham. *p < 0.001* | JMT produced a statistically significant increase in BR compared to Sham. *p = 0.006* | | | JMT produced a statistically significant increase in HR compared to Sham *p < 0.001* | | No statistically significant difference |  |
| Yung  2014 [46] | C6 antero-posterior mobilization at 1.5 Hz | - Baseline (at 5 and 7 min of rest  - Intervention (during the first and fifth sets)  - Post Intervention (2 min after the fifth set) | - BP  - HR | ? | ***HR post intervention*** | | ***SBP post intervention*** | | | ***HR, DBP, SBP*** | | The between-group differences were not clearly reported. |
|  |  |  |  |  | JMT produced a statistically significant decrease in HR from baseline to post intervention. No significant difference in the Sham group | | Both JMT and Sham produced a significant decrease in SBP from baseline to post intervention. | | | No other significant difference. | |  |
| BP: blood pressure; BR: breathing rate; HR: heart rate; SBP: systolic blood pressure; SC: skin conductance; ST: skin temperature; DBP: diastolic blood pressure | | | | | | | | | | | | |

| **Results table**  **Mobilizations (oscillatory technique**) | | | | | | | | | | |
| --- | --- | --- | --- | --- | --- | --- | --- | --- | --- | --- |
| **Study ID** | **Joint manipulative technique** | **Measurement period**  **(duration)** | **Outcomes** | **Statistical analysis: statistical tests,** **Level** | **Summary of the results**  **(Comparison between JMT and Sham)** | | | | **Other findings**  **and comments** | |
| Piekarz  2016 [47] | 1. L4 3 Hz Maitland mobilization  2. L4 2 Hz Maitland mobilization | - Baseline (2 min)  - Intervention (5 min)  - Post intervention (5 min) | SC « 1-min integral measurement »  (% change from baseline) | - GLM (Time factor, intervention factor)  - Post Hoc analysis with Bonferroni corrections  - Alpha: 0.05 | ***SC intervention*** | | ***SC post intervention*** | | - No statistically significant difference between the 3 Hz and 2 Hz JMT  - No statistically significant difference between Sham and Control | |
|  |  |  |  |  | 1. JMT produced a statistically significant increase in SC (20.1% ± 5.38) compared to Sham (-1.3% ± 0.74). *p = 0.002* | | 1. No statistically significant difference | |  |  |
|  |  |  |  |  | 2. No statistically significant difference.  JMT produced an increase in SC (12.4% ± 4.77) compared to Sham (-1.3% ± 0.74).  *p = 0.095* | | 2. No statistically significant difference | |  |  |
| Zegarra-Parodi  2016 [48] | 1. T1 High pressure mobilization (80% of the PPT) 0.5 Hz  2. T1 Low pressure mobilization (40% of the PPT) 0.5 Hz | - Baseline (3 min)  - Intervention (5 min)  - Post intervention (5 min) | - SBF (arbitrary perfusion unit)  - ST  - HR  - Mean arterial blood pressure | - Linear mixed model (within- and between- group differences)  - Alpha: 0.004 (within-group) and 0.05 | ***SBF intervention and post intervention*** | | ***ST, MAP, HR intervention and / or post intervention*** | | Sham, low pressure JMT, High pressure JMT significantly increased SBF during the second half of JMT application. The “effect” was transient for sham and low pressure JMT but remained significant between sets for high pressure JMT. The response was attenuated after the third set of JMT in the three pressure interventions. There was a decrease in SBF during the inspiratory gasp.  There was a statistically significant difference in SBF (14.7 %; 95 % CI, 2.9-26.6%) between Sham and Control  Results were similar on the contralateral side for SBF |  |
|  |  |  |  |  | 1. No statistically significant difference.  *p* = 0.22 | | 1. No statistically significant difference | |  |  |
|  |  |  |  |  | 2. No statistically significant difference.  *p = 0.22* | | 2. No statistically significant difference | |  |  |
| Yung  2017 [49] | C6 postero-anterior mobilization at 1.5 Hz | - Baseline (at 5 and 7 min of rest  - Intervention (during the first and fifth sets)  - Post Intervention (2 and 4 min after the fifth set) | - BP  - HR | - Multilevel mixed effect modelling for repeated measures  - *t* test with Bonferroni adjustment  -Alpha: 0.05 | ***SBP intervention*** | ***SBP post intervention*** | | ***HR, DBP intervention and post intervention*** |  |  |
|  |  |  |  |  | JMT produced a statistically significant decrease in SBP (-2.95 ± 3.11) compared to Sham (-0.72 ± 4.66). *p = 0.003* | No statistically significant difference | | No statistically significant difference |  |  |
| Araujo  2017 [50] | 1. T4 Grade III posterior-to-anterior rotatory passive accessory intervertebral mobilization at 2 Hz  2. T4 unilateral grade III posterior-to-anterior rotatory passive accessory intervertebral mobilization at 2 Hz + slump | - Baseline  - Post Intervention (5 min blocks within 15 min) | - Mean RR  - HR  - STD HR  - SDNN  - RMSSD  - RRtrindex  - LF/HF  - normalized power of HF and LF bands | - Univariate ANCOVA  - Pairwise comparisons with Bonferroni adjustment for multiple  comparisons  - Alpha: 0.05 | ***Mean RR, HR, STD HR, SDNN, RMSSD, Rr trindex, LF/HF, normalized power of HF, normalized power of LF*** | | | |  |  |
|  |  |  |  |  | 1. No statistically significant difference | | | |  |  |
|  |  |  |  |  | 2. No statistically significant difference | | | |  |  |
| HR: heart rate; SBP: systolic blood pressure; DBP: diastolic blood pressure; GLM: general linear model; HF: high frequency; HR: heart rate; JMT: joint manipulative technique; LF: low frequency; MAP: mean arterial blood pressure; Mean RR: mean RR interval; PPT: pressure pain threshold; RMSSD: square root of the mean of the sum of the squares of differences between adjacent NN intervals; Rrtrindex: RR triangular index; SBF: skin blood flow; SDNN: standard deviation of NN intervals; SC: skin conductance; ST: skin temperature; STD HR: heart rate standard deviation; | | | | | | | | | |  |

| **Results table**  **Mobilizations (atypical technique)** | | | | | | | | |
| --- | --- | --- | --- | --- | --- | --- | --- | --- |
| **Study ID** | **Joint manipulative technique** | **Measurement period**  **(duration)** | **Outcomes** | **Statistical analysis: Statistical test**  **Level** | **Summary of the results**  **(Comparison between JMT and Sham)** | | | **Other findings**  **and comments** |
| Henderson  2010 [51] | Rib raising mobilization  T1-T4 ; T5-T8 ; T9-T12 | -Baseline  -Immediately after intervention  -10 min after intervention | - salivary α-amylase  - salivary flow rate | -ANOVA (intervention factor, time factor)  - *t* test (pre / post)  - Alpha : 0.05 | ***salivary α-amylase (immediately after intervention)*** | ***salivary α-amylase (10min)*** | ***salivary flow rate*** |  |
|  |  |  |  |  | JMT produced a statistically significant decrease in α-amylase compared to Sham. *p = 0.046* | JMT produced a statistically significant decrease in α-amylase compared to Sham.  *p = 0.046* | No statistically significant difference |  |
|  |  |  |  |  |  |  |  |  |

| **Results table**  **Mobilizations (SNAGs / mobilization with movement)** | | | | | | | | | | | | | |
| --- | --- | --- | --- | --- | --- | --- | --- | --- | --- | --- | --- | --- | --- |
| **Study ID** | **Joint manipulative technique** | **Measurement period**  **(duration)** | **Outcomes** | **Statistical analysis: statistical tests,** **Level** | **Summary of the results**  **(Comparison between JMT and Sham)** | | | | | | | | **Other findings**  **and comments** |
| Paungmali  2003 [52] | Elbow mobilization with movement  *10 repetitions sustained 6 secs with 15 secs at rest between* | -Baseline (2 min)  - Intervention  - Post intervention | Maximum value of:  - SC  - SBF  - ST  - BP  - HR | - ANOVA (side factor for SC and ST, intervention factor, time factor)  - Post hoc analysis *t* tests with Bonferroni corrections  - Alpha: 0.0083 and 0.017 | ***SC MAX intervention*** | ***ST MAX and MIN intervention*** | | ***SBF MAX and MIN intervention*** | | ***HR MAX post intervention*** | | ***BP MAX post intervention*** | No change for both the Sham and Control interventions |
|  |  |  |  |  | JMT produced a statistically significant increase in SC on the affected side (55.0 %) compared to Sham (no change).  Treatment by time interaction  *p = 0.002* | JMT produced a statistically significant decrease in hand skin temperature (–1.1 %) and a statistically significant increase in elbow skin temperature (2.1 %) on the affected side compared to Sham (no change).  Treatment by time interaction  *p = 0.001* | | JMT produced a statistically significant decrease in hand blood flux (–72.4 %) and a statistically significant increase in elbow blood flux (123.7%) on the affected side compared to Sham (no change.  Treatment by time interaction  *p = 0.001* | | JMT produced a statistically significant increase in HR (4.1 %) compared to Sham (no change).  Treatment by time interaction  *p = 0.001* | | JMT produced a statistically significant increase in SBP (3.5%) and DBP (3.1 %) compared to Sham (no change).  Treatment by time interaction  *p = 0.001* |  |
| Moulson  2006 [53] | C5/C6 Mulligan’s sustained natural apophyseal glides with active head rotation | -Baseline (2 min)  -Intervention  -Post intervention (2 min) | - SC  - ST  (Difference A between intervention and baseline; difference B between post intervention and baseline) | - ANOVA (Side factor, intervention factor)  - Post Hoc analysis with Bonferroni correction  - Alpha: ≤ 0.05 | ***SC intervention period (diff A)*** | | ***SC post intervention period (diff B)*** | | | | ***ST Diff A and B*** | | No side difference  Statistically significant difference between sham and control for SC  No statistically significant difference between sham and control for ST |
|  |  |  |  |  | No statistically significant difference | | JMT produced a statistically significant increase in SC (0.140; 95 % CI [0.061; 0.220]) compared to Sham (0.032; 95 % CI [0.008; 0.056]). *p = 0.010* | | | | No statistically significant difference | |  |
| Moutzouri  2012 [54] | L4 Mulligan’s sustained natural apophyseal glides while participant performed 6 repetitions of full active lumbar flexion in sitting | -Baseline (3 min)  -Intervention (3 min)  - Post intervention (3 min) | - SC integral measurement  (% change from baseline) | - ANOVA (intervention factor)  - Post Hoc analysis with Bonferroni corrections  -*t* test (side comparison)  - Alpha: 0.05 | ***SC intervention period*** | | | | ***SC post intervention period*** | | | | No side difference  No statistically significant difference between sham and control for both limbs. |
|  |  |  |  |  | No statistically significant difference | | | | No statistically significant difference | | | |  |
| Tsirakis  2015 [55] | L4 Mulligan’s sustained medial glides with passive right leg flexion movements (repeated 3 times) | - Baseline (2 min)  - Intervention  - Post Intervention (2 min) | SC « integral measurement »  (% change from baseline) | - ANOVA (intervention factor, Time factor, Side factor)  - Post Hoc analysis with Bonferroni corrections  - Alpha: 0.05 | ***SC treatment period*** | | | | ***SC post treatment period*** | | | | No statistically significant difference between sham and control |
|  |  |  |  |  | No statistically significant difference | | | | No statistically significant difference | | | |  |
| Bowler  2017 [56] | C5 right (ipsilateral technique) or left (contralateral technique) articular pillar | - Baseline (2min)  - Intervention  - Post intervention (2 min) | - SC  (% change from baseline)  - ST | - ANOVA  - Post Hoc analysis with Bonferroni corrections  - Alpha: 0.05 | **SC intervention and post intervention periods** | | | | **ST intervention and post intervention periods** | | | | There was a statistically significant difference between sham and control for SC. |
|  |  |  |  |  | No statistically significant difference | | | | No statistically significant difference | | | |  |

| **Results table**  **HVLA manipulation** | | | | | | | | | |
| --- | --- | --- | --- | --- | --- | --- | --- | --- | --- |
| **Study ID** | **Joint manipulative technique** | **Measurement period**  **(duration)** | **Outcomes** | **Statistical analysis: statistical tests,** **Level** | **Summary of the results**  **(Comparison between JMT and Sham)** | | | | **Other findings**  **and comments** |
| Budgell  2001 [57] | C1/C2 HVLA manipulation | - Baseline (5min)  - Post intervention (5min) | - Power of the absolute and normalized LF component  - Power of the absolute and normalized HF component  - LF/HF  - HR | - *t* test or Wilcoxon signed rank test (pre / post comparison)  - Alpha: 0.05 | **Power of the absolute and normalized LF component** | **Power of the absolute and normalized HF component** | **LF/HF** | **HR** | The between-group differences were not reported. |
|  |  |  |  |  | JMT produced a statistically significant increase in the power of the absolute and the normalized LF component. p < 0.05  Sham produced no statistically significant change | No statistically significant change for both JMT and sham | JMT produced a statistically significant increase in the ratio LF/HF. *P = 0.0037*  Sham produced no statistically significant change | Both JMT and sham produced a statistically significant decrease in HR |  |
| Budgell  2006 [58] | T1-T4 HVLA manipulation | - Baseline (5min)  - Post intervention (5min) | - Power of the absolute and normalized LF component  - Power of the absolute and normalized HF component  - LF/HF  - HR | - *t* test or Wilcoxon signed rank test (pre / post comparisons)  - Alpha: 0.05 | **Power of the absolute and normalized LF component** | **Power of the absolute and normalized HF component** | **LF/HF** | **HR** | The between-group differences were not reported. |
|  |  |  |  |  | JMT produced a statistically significant increase in the power of the absolute and the normalized LF component.  *p < 0.05.*  Sham produced no statistically significant change | JMT produced a statistically significant decrease in the power of the normalized HF component  *p = 0.0043.*  Sham produced no statistically significant change. | JMT produced a statistically significant increase in the ratio LF/HF. p < 0.05  Sham produced no statistically significant change | Both JMT and sham produced a statistically significant decrease in HR |  |
| Roy  2009 [59]  (only the pain group) | L5 HVLA manipulation | - Baseline (5 min)  - Post intervention (5 min) | HRV:  - Mean RR  - SDNN  - NN 50 count  - pNN 50 %  - VLF  - Power of the LF component  - Power of the HF component  - LF/HF | - ANOVA  - Post Hoc analysis with Tukey HSD  - Alpha: 0.05 | **Mean RR, SDNN, NN 50 count, pNN 50 %, VLF, power of the LF component, power of the HF component, LF/HF** | | | | - For the spectral power in the VLF and HF band there was a significant between-subject effect but no within-subject effect and no groups by times interaction.  - Spectral power (normalized unit) in the VLF band increased in both Treatment (*d,* 0.36) and Sham (*d*, 0.34) groups  - Spectral power (normalized unit) in the HF band decreased in both Treatment (*d,* 0.06) and Sham (*d*, 0.21) groups |
|  |  |  |  |  | No statistically significant change | | | |  |
| *d*: Cohen’s d; HRV: heart rate variability; HF: high frequency; LF: low frequency; NN 50 count: successive N–N intervals differing more than 50 ms; pNN 50%: The proportion of NN50 divided by the total number of NN (R-R) intervals; SDNN: standard deviation of NN intervals; VLF: very low frequency | | | | | | | | | |

| **Results table**  **HVLA manipulation** | | | | | | |  |
| --- | --- | --- | --- | --- | --- | --- | --- |
| **Study ID** | **Joint manipulative technique** | **Measurement period**  **(duration)** | **Outcomes** | **Statistical analysis: statistical tests,** **Level** | **Summary of the results**  **(Comparison between JMT and Sham)** | **Other findings**  **and comments** |  |
| Sillevis  2010 [60] | T3 / T4 HVLA manipulation | - Baseline (1min)  - Post intervention 1 (1min)  - Post Intervention 2 + 4 min (1min) | - Pupil diameter (pixels) | - Friedman’s test (pre / post comparisons)  - Wilcoxon signed rank test (comparison between groups) | **Pupil diameter** |  | |
|  |  |  |  |  | No statistically significant change (pre / post) in the treatment group |  |  |
| Puhl  2012 [61] | T1-T6 HVLA manipulation | - Baseline  - Post intervention 1 immediately  - Post intervention 2 + 15 min | Plasma level of:  - Norepinephrine  - Epinephrine | -*t* test (pre / post comparisons and group comparisons)  - Alpha: 0.05 | **Plasma level of norepinephrine and epinephrine at any time point** |  | |
|  |  |  |  |  | No statistically significant change (pre / post) for both treatment and sham groups |  |  |
| Ward  2013 [62] | T1-T4 HVLA manipulation | - Baseline (10s)  - Post intervention 1 +1 min (10 secs)  - Post Intervention 2 +10 min (10 secs)  - Post 24 hours | -HR  -BP | - ANOVA (group factor)  - Post Hoc analysis with Bonferroni  - *t* test (pre / post comparisons)  - Alpha: 0.05 | **HR, BP** | No statistically significant difference between sham and control | |
|  |  |  |  |  | No statistically significant change (within-group and between-groups) |  |  |
| Sampath  2017 [63] | T5 HVLA manipulation | - Baseline (5 min)  - Post intervention time point  HRV: 5 min and 30 min (5 min blocks)  Oxy-hemoglobin: 1 min, 5 min and 30 min | - HRV (LF/HF)  - Oxy-hemoglobin | - ANOVA (time factor, intervention factor)  - Post Hoc analysis with Bonferroni corrections  - Alpha: 0.05 | **HRV (LF/HF), Oxy-hemoglobin concentration** |  | |
|  |  |  |  |  | No statistically significant difference |  |  |
| BP: blood pressure; HF: power of the high frequency component; HR: heart rate; LF: power of the low frequency component | | | | | | | |
